# An unanticipated tumor-suppressive role of the SUMO pathway in the intestine unveiled by Ubc9 haploinsufficiency

**DOI:** 10.1038/s41388-020-01457-y

**Published:** 2020-09-18

**Authors:** Ignacio López, Eleftheria Chalatsi, Saskia I. J. Ellenbroek, Alexandra Andrieux, Pierre-François Roux, Juan P. Cerapio, Grégory Jouvion, Jacco van Rheenen, Jacob-S. Seeler, Anne Dejean

**Affiliations:** 1 Nuclear Organization and Oncogenesis Unit, INSERM U993, Equipe Labellisée Ligue Nationale Contre le Cancer, Institut Pasteur, 75015 Paris, France; 2 Collège Doctoral, Sorbonne Université, 75005 Paris, France; 3 Division of Molecular Pathology, Oncode Institute, The Netherlands Cancer Institute, Amsterdam, The Netherlands; 4 Experimental Neuropathology Unit, Institut Pasteur, 75015 Paris, France; 5 Present Address: Bio-Rad Laboratories, Marnes-la-Coquette, France; 6 Present Address: Centre de Recherches en Cancérologie de Toulouse, Université de Toulouse, Toulouse, France

**Keywords:** Colorectal cancer, Sumoylation

## Abstract

Sumoylation is an essential posttranslational modification in eukaryotes that has emerged as an important pathway in oncogenic processes. Most human cancers display hyperactivated sumoylation and many cancer cells are remarkably sensitive to its inhibition, thus supporting application of chemical sumoylation inhibitors in cancer treatment. Here we show, first, that transformed embryonic fibroblasts derived from mice haploinsufficient for Ubc9, the essential and unique gene encoding the SUMO E2 conjugating enzyme, exhibit enhanced proliferation and transformed phenotypes in vitro and as xenografts ex vivo. To then evaluate the possible impact of loss of one *Ubc9* allele in vivo, we used a mouse model of intestinal tumorigenesis. We crossed *Ubc9*^*+/−*^ mice with mice harboring a conditional ablation of Apc either all along the crypt–villus axis or only in Lgr5^+^ crypt-based columnar (CBC) cells, the cell compartment that includes the intestinal stem cells proposed as cells-of-origin of intestinal cancer. While *Ubc9*^*+/−*^ mice display no overt phenotypes and no globally visible hyposumoylation in cells of the small intestine, we found, strikingly, that, upon loss of Apc in both models, *Ubc9*^*+/−*^ mice develop more (>2-fold) intestinal adenomas and show significantly shortened survival. This is accompanied by reduced global sumoylation levels in the polyps, indicating that Ubc9 levels become critical upon oncogenic stress. Moreover, we found that, in normal conditions, *Ubc9*^*+/−*^ mice show a moderate but robust (15%) increase in the number of Lgr5^+^ CBC cells when compared to their wild-type littermates, and further, that these cells display higher degree of stemness and cancer-related and inflammatory gene expression signatures that, altogether, may contribute to enhanced intestinal tumorigenesis. The phenotypes of Ubc9 haploinsufficiency discovered here indicate an unanticipated tumor-suppressive role of sumoylation, one that may have important implications for optimal use of sumoylation inhibitors in the clinic.

## Introduction

SUMO modification has emerged as an essential and highly dynamic posttranslational modification (PTM) that targets hundreds of cellular proteins (the “sumoylome”), thus affecting most, if not all, fundamental processes carried out by eukaryotic cells [1, 2]. As with other, “small-molecule” PTMs (phosphorylation, acetylation, methylation, etc.), the covalent attachment of the ~11 KDa SUMO peptide changes the interacting properties of its target proteins and hence their functions as parts of larger molecular complexes or pathways.

Numerous stresses and pathological conditions, notably infection and cancer, result in the rapid modification, but also de-modification, of numerous substrates with, at times, profound effects on the composition of the cellular sumoylome [2–7]. Conversely, strong perturbation of the SUMO system by inhibiting the activity of the unique E1 (SAE1/SAE2) or E2 (Ubc9) enzymes, for example, provokes significant cell stress that leads to growth arrest, senescence or apoptosis [8–11].

As with stress, the cancerous state is associated with dysregulated cellular sumoylation dynamics stemming from the altered, mostly upregulated expression of SUMO pathway components (i.e., SUMO, E1, E2, and SENPs) that affects numerous SUMO-targeted cellular factors, including key tumor suppressors and oncoproteins [6, 7]. The concept of “non-oncogene addiction” [12, 13] has been suggested as a possible view of SUMO’s roles in cancer: the idea that with strong oncogenic signaling, for example by elevated Myc or Ras activity, the stress-mitigating functions of sumoylation become indispensable for cancer cell survival [14–16]. Clearly, this makes SUMO modification an attractive target for cancer therapy, particularly in cases such as Myc-driven cancers in which the oncoprotein itself remains out of range.

Our understanding of the origins of colorectal cancer (CRC) has greatly benefited from a large body of work, including elegant lineage tracing studies in mice [17]. These studies provided a detailed spatial and temporal description of the rapid, continuous and orderly developmental progression that links the Lgr5^+^ intestinal stem cell, Paneth and transit-amplifying cell compartment with the differentiated epithelial cells to constitute the functional crypt–villus anatomy of the small intestinal epithelium. Ninety percent of CRC cases follow a well-defined and ordered chain of genetic events starting with perturbation of APC/β-catenin signaling followed by dysregulation of the KRAS, TP53, and/or PIK3CA pathways [18–20]. Significantly, the progression from normal intestinal mucosa, through aberrant crypt foci, small intestinal adenomas or polyps to malignant tumors (but rarely to colonic tumors) is recapitulated in mouse models for the specific and inducible ablation of the Apc tumor suppressor [20]. The characterization of Lgr5^+^ crypt-based columnar (CBC) cells, that include the intestinal stem cells (ISCs) shown to act as cells-of-origin of intestinal cancer [21, 22], attests to the value of these models.

We have shown previously that complete loss of Ubc9, and hence of sumoylation, is early embryonic lethal in mice [10]. Similarly, acute loss of Ubc9 in adult mice leads to rapid death caused by degeneration of the intestinal epithelium [23]. In this setting, lack of sumoylation primarily affects the crypt compartment by causing the disappearance of the Lgr5^+^ CBC cells that leads to rapid disorganization of the crypt–villus axis, thereby severely compromising the genesis, function, positioning and survival of all differentiated epithelial cells. Since sumoylation is essential for cell and animal viability, we analyzed here the role of the SUMO pathway in cancer-related processes using in vitro and in vivo Ubc9 haploinsufficient models. Loss of a single *Ubc9* allele in transformed fibroblasts conferred enhanced proliferative and transformed phenotypes. In addition, while without any major phenotypic effects in mice in normal conditions, Ubc9 haploinsufficiency was found to enhance intestinal tumorigenesis and to provoke earlier death in Apc-deficient backgrounds. Furthermore, we found this phenotype to be associated with both increased numbers of crypt-based Lgr5^+^ CBC cells and exacerbated stemness, inflammatory and cancer-related gene expression programs in this cellular context. These results uncover an unexpected oncosuppressive role of sumoylation with potential impact for cancer treatment.

## Results

### Ubc9 haploinsufficiency promotes cell growth and transformed phenotypes in vitro and ex vivo

**Fig. 1:**
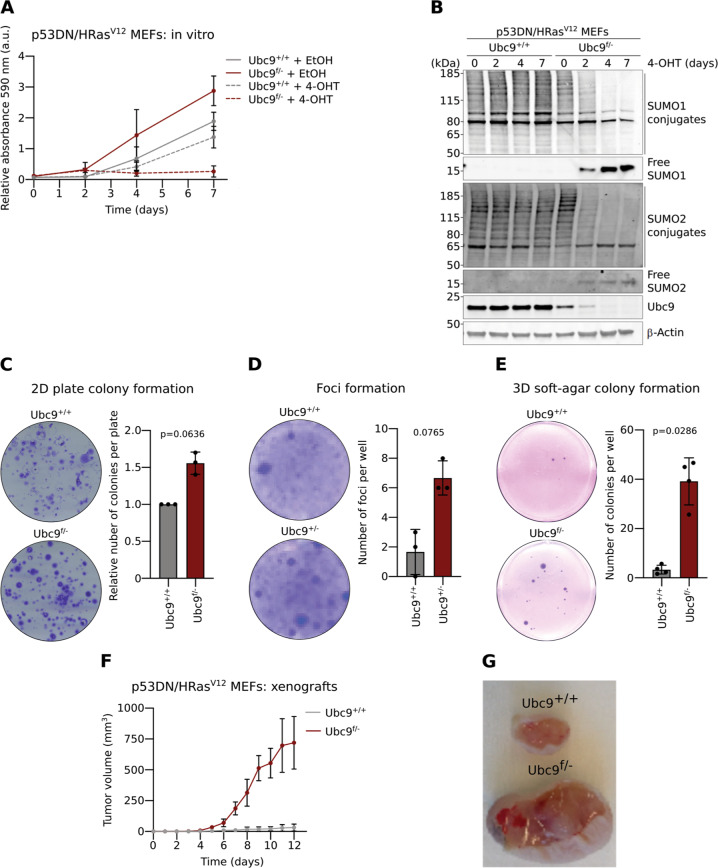
*Ubc9* haploinsufficiency promotes cell growth and malignant phenotypes in vitro. **a** Growth curve of p53DN/HRAS^V12^-transformed MEFs derived from 3 pooled embryos *Ubc9*^*+/+*^ and *Ubc9*^*f/−*^ *Rosa26-Cre*^*ERT2*^ treated with 100 μg/mL 4-OHT or EtOH (control). 590 nm absorbance of cell-trapped crystal violet was measured to estimate relative cell numbers. Mean ± SD, *n* = 3 biological replicates. **b** Western-blotting showing SUMO1 and SUMO2 conjugates and Ubc9 expression in *Ubc9*^*+/+*^ and *Ubc9*^*f/*−^ *Rosa26-Cre*^*ERT2*^ MEFs treated with 100 μg/mL 4-OHT for 0, 2, 4, and 7 days. **c** Capacity of transformed MEFs for anchorage-dependent 2D plate colony formation. Representative images and quantification showing mean ± SD. *n* = 3 biological replicates in duplicates. Unpaired two-tailed Mann–Whitney test was used. **d** Capacity of transformed MEFs for loss of contact inhibition and foci formation, as in (**c**), with *n* = 3 biological replicates. **e** Capacity of transformed MEFs for anchorage-independent 3D soft-agar colony formation, as in (**c**), with *n* = 4 biological replicates in triplicates. **f** Growth curve of p53DN/HRAS^V12^-transformed MEF xenografts in mice. Mean ± SD, *n* = 6 mice injected with *Ubc9*^*f/*–^ and *Ubc9*^*+/+*^ transformed MEFs in opposite flanks of each animal. **g** Representative picture of tumors, as in (**f**), taken from the same mouse 12 days after inoculation.

To address the role of Ubc9 in controlling cell proliferation and tumorigenesis, we used mice haploinsufficient for Ubc9 which harbour a *floxed* and a *null Ubc9* allele (*Ubc9*^*f/*−^) [23]. While *Ubc9*^−*/−*^ animals are embryonic lethal, *Ubc9*^*f/−*^, like *Ubc9*^*+/−*^ mice display no overt phenotype in normal conditions, apart from a slight (10%) decrease in body size and body weight, when compared to wild-type (WT) littermates [10]. Murine embryo fibroblasts (MEFs) derived from a pool of three E12.5 WT or *Ubc9*^*f/−*^ embryos were transformed by retroviral transduction of a dominant-negative (DN) p53 mutant together with the HRAS^V12^ oncogene. *Ubc9*^*f/−*^ MEFs were found to grow significantly faster than *Ubc9*^+/+^ MEFs and inducible deletion of the remaining *floxed Ubc9* allele by 4-hydroxytamoxifen (4-OHT) [23] expectedly abrogated their proliferation capacity (Fig. 1a). This pattern correlated with suppression of Ubc9 protein expression, reduction in global sumoylation and appearance of free SUMO1 and SUMO2/3 (hereafter SUMO2−) (Fig. 1b).

To further characterize the proliferative advantage of non-treated *Ubc9*^*f/−*^ over *Ubc9*^+/+^ transformed MEFs, we analyzed cell cycle distribution and apoptosis rates at 4 days post-passage, when cells are still non-quiescent but the difference in cell numbers is clearly evident (Fig. S1a). While the cell cycle distribution was very similar for cells of both genotypes (Fig. S1b), we found a slightly larger fraction of apoptotic cells in *Ubc9*^*+/+*^ (mean 9.8%) compared to *Ubc9*^*f/−*^ (6.7%) cells (Fig. S1c), a difference, however, likely insufficient to fully explain the observed, significant 1.7-fold difference in cell numbers after 4 days’ growth. To next see whether the growth difference could be related to the levels of the exogenous oncogenes, we compared the expression of p53 and HRAS between both genotypes. RT-qPCR analysis showed that levels of endogenous *p53* and *HRas* are similar in both contexts (Fig. S1d). Interestingly, however, expression of exogenous murine *p53DN* and human *HRAS*^*V12*^ mRNA was higher in *Ubc9*^*+/+*^ cells (Fig. S1e), a finding also confirmed for HRAS proteins by Western blot (Fig. S1f). Together, this suggests that the increased proliferation capacity displayed by mutant MEFs is not driven by higher oncogene activity but might rather be a consequence of a more transformation-prone basal state in Ubc9 haploinsufficient MEFs.

The ability of transformed cells to form colonies of clonal origin on plates and in agar, or to form foci when seeded at high densities, is related to their capacity to grow and divide in an anchorage-dependent and anchorage-independent manner, or to lose contact inhibition, respectively [24–26]. Transformed *Ubc9*^*f/−*^ MEFs formed colonies and foci more efficiently than their WT counterparts, as seen by the 1.6-, 4- and 12-fold growth increase in 2D plate colony-, foci formation and 3D soft-agar colony assays, respectively (Fig. 1c–e). To transpose these findings in vivo, we xenografted nude mice with transformed *Ubc9*^+/+^ and *Ubc9*^*f/−*^ MEFs and found that Ubc9 haploinsufficiency greatly enhanced tumor growth, consistent with the findings obtained in vitro (Fig. 1f–g). Altogether, these data suggest that mild Ubc9 downregulation confers a growth advantage consistent with the establishment of an exacerbated transformed state.

### Ubc9 haploinsufficiency favors polyp formation in an *Apc*^*f/+*^ intestinal cancer mouse model

**Fig. 2:**
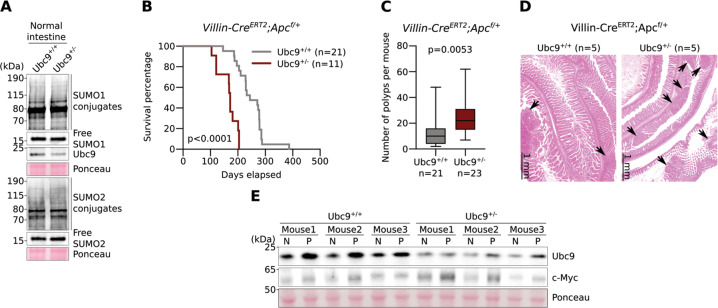
*Ubc9* haploinsufficiency favors polyp formation in *Villin-Cre*^*ERT2*^*;Apc*^*f/+*^ intestinal cancer mouse model. **a** Western-blotting of SUMO1 and SUMO2 conjugates and Ubc9 in intestines from healthy mice. **b** Kaplan–Meier survival curves of *Ubc9*^*+/+*^ and *Ubc9*^*+/−*^ *Villin-Cre*^*ERT2*^*;Apc*^*f/+*^ mice treated with 4-OHT (0.1 mg per g of body weight). Log-rank test was used. **c** Median with min, 25 and 75 percentiles, and max number of small intestinal adenomas (≥0.5 mm in diameter) in *Ubc9*^*+/+*^ and *Ubc9*^*+/−*^ mice 12 weeks after treatment with 4-OHT as in (**b**). Unpaired two-tailed *t* test was used. **d** Histological analysis of hematoxylin and eosin (H&E)-stained small intestinal sections obtained as in (**c**). Arrows indicate lesions. **e** Western-blotting of Ubc9 and c-Myc in polyps (P) and normal (N) intestine samples obtained as in (**c**) in three different mice per group.

We next investigated the effect of Ubc9 haploinsufficiency on tumor initiation and development in vivo, using the *Apc*^*f/+*^ intestinal cancer mouse model. First, and in agreement with the situation in WT and *Ubc9*^*f/−*^ MEFs (compare “0” lanes in Fig. 1b), we found that global profiles of SUMO-conjugated proteins in whole intestinal extracts from WT and *Ubc9*^*+/−*^ mice are indistinguishable (Fig. 2a), although this does not exclude that effects on specific substrates go undetected by this global analysis. Of note, the Ubc9 protein level was reduced to about a half of normal levels in *Ubc9*^*f/−*^ intestines, indicating the absence of compensatory mechanisms for Ubc9 expression in these animals (Fig. 2a). Furthermore, anatomopathological analysis of intestines from mice of both genotypes did not reveal significant differences in the general architecture of crypts (Fig. S2a).

Next, we generated *Ubc9;Villin-Cre*^*ERT2*^*;Apc*^*f/+*^ mouse strains in which Cre^ERT2^ recombinase expression is driven by the intestine-specific *Villin* promoter [27, 28]. After Cre^ERT2^ activation by intraperitoneal injections of 4-OHT, the *lox*P-flanked exon 14 in one allele of the *Apc* tumor suppressor is deleted all along the crypt–villus axis leading to the initial stage of the multi-step transformation process [19, 20] in either the WT or *Ubc9*^*+/−*^ genetic background. Surprisingly, we found that 4-OHT treatment resulted in shortened survival of *Ubc9*^*+/−*^ mice compared to their *Ubc9*^+/+^ counterparts (Fig. 2b). To determine whether this effect was related to tumor incidence and/or load, we assessed the number of adenomatous polyps (≥0.5 mm in diameter) generated 12 weeks after 4-OHT treatment. We found that *Ubc9*^*+/−*^ mice developed twofold more adenomas, with medians of 10 and 22 polyps per animal in small intestines of *Ubc9*^*+/+*^ and *Ubc9*^*+/−*^ mice, respectively (Figs. 2c, S2b). Hematoxylin and eosin (H&E) staining of these samples further confirmed the increased frequency of both dysplasia and tumors in *Ubc9*^*+/−*^ mice (Figs. 2d, S2c). No differences in the morphology and general organization of tumors and dysplasia foci were found (Figs. 2d, S2b–d). Finally, the median size of *Ubc9*^*+/−*^ polyps appears to be half that of WT (Fig. S2e), although, due to rounding off the measurements to the nearest 1 mm increment, the real median size difference may likely be less pronounced. A control experiment to address the possibility that Ubc9 haploinsufficiency merely accelerates Apc loss, and hence tumorigenesis, revealed that reduction of Ubc9 levels, here carried out in human U2OS cells, likely does not affect homologous recombination (HR), a process strictly required for tumorigenesis by loss of the second, WT Apc allele in the intestines of *Ubc9;Villin-CreERT2;Apc*^*f/+*^ mice (Fig. S2h–j). This suggests that the higher adenoma frequency seen in *Ubc9*^*+/−*^ mice is unlikely due to enhanced HR-dependent inactivation of the native *Apc* allele. Together, these data indicate that decreasing Ubc9 levels to a half-dose promotes intestinal tumorigenesis. Moreover, they suggest that tumor initiation, rather than development, is enhanced in this process.

### *Ubc9*^*+/+*^ and *Ubc9*^*+/−*^ mice display similar transcriptomic profiles in adenomatous polyps or in normal intestinal tissue

We next sought to pinpoint the molecular pathways responsible for the stimulated intestinal tumorigenesis observed in *Ubc9*^*+/−*^ mice. For this, we first compared Ubc9 protein levels in normal (N) and polyp (P) intestinal samples from *Villin-Cre*^*ERT2*^*;Apc*^*f/+*^ mice 12 weeks after 4-OHT treatment. Higher c-Myc levels confirmed the transformed nature of polyp samples from both *Ubc9*^*+/+*^ and *Ubc9*^*+/−*^ genotypes (Fig. 2e). Besides the expected reduction in *Ubc9*^*+/−*^ samples, interestingly, Ubc9 levels were nevertheless higher in polyps than in adjacent normal tissue in both the WT and the *Ubc9*^*+/−*^ contexts (Fig. 2e).

Transcriptomic analysis of dissected polyps as well as of neighboring normal intestinal tissues from these animals revealed a total of only 11 differentially-expressed genes (DEGs, |log2FC| > log2(1.5) and adj. *p* value (FDR) < 0.05), with 7 and 4 up- and downregulated, respectively, in *Ubc9*^*+/−*^ polyps when compared to WT (Fig. S2f, Table S1). Similar analysis of the neighboring normal intestinal tissue yielded only a set of 31 DEGs, of which 8 and 23 were up- and downregulated, respectively, in the *Ubc9*^*+/−*^ animals (Fig. S2g, Table S2). Gene Ontology (GO) analysis applied to these small lists of genes failed to discern any specific ontology term related to cancer development, suggesting that *Ubc9* status has little or no effect on the specific gene expression signatures of polyps or normal intestinal tissue, or that analysis of these “bulk” tissues may have failed to unmask cell type-specific transcriptional differences.

### Ubc9 haploinsufficiency increases tumorigenesis upon Apc loss in Lgr5^+^ CBC cells

**Fig. 3:**
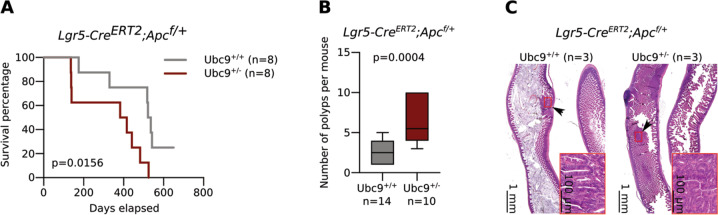
*Ubc9* haploinsufficiency favors polyp formation in *Lgr5-IRES-EGFP-Cre*^*ERT2*^*;Apc*^*f/+*^ intestinal cancer mouse model. **a** Kaplan–Meier survival curves of *Ubc9*^*+/+*^ and *Ubc9*^*+/−*^ *Lgr5-IRES-EGFP-Cre*^*ERT2*^*;Apc*^*f/+*^ mice treated with 4-OHT as in Fig. 1b. Log-rank test was used. **b** Median number of small intestinal adenomas (≥0.5 mm in diameter) with min, 25 and 75 percentiles, and max values in *Ubc9*^*+/+*^ and *Ubc9*^*+/−*^ mice 16 weeks after treatment with 4-OHT, as in Fig. 1a. Unpaired two-tailed Mann–Whitney test was used. **c** Histological analysis of H&E-stained small intestinal sections obtained as in (**b**). Arrows indicate lesions also shown in the magnified insets.

Given the enhancing effect of reduced Ubc9 levels on intestinal tumorigenesis, yet the absence of a clear transcriptomic effect in either bulk polyps or normal intestine, we focused subsequent analyses on the more restricted set of CBC cells that includes early progenitors, and importantly, the intestinal stem cells [22]. For this, we generated *Ubc9;Lgr5-IRES-EGFP-Cre*^*ERT2*^*;Apc*^*f/+*^ mouse strains in which deletion of one *lox*P-flanked *Apc* allele is restricted to CBC cells [21]. In line with previous results (Fig. 2b), *Ubc9*^*+/−*^ 4-OHT-treated mice displayed shorter survival than their *Ubc*^*+/+*^ counterparts (Figs. 3a, S3a). Moreover, mutant mice developed >2-fold more adenomas (≥0.5 mm in diameter), albeit with smaller size, in the small intestine 16 weeks after 4-OHT treatment (Figs. 3b, S3b). It is noteworthy that transformation rates in *Lgr5-IRES-EGFP-Cre*^*ERT2*^ mice were approximately threefold lower than previously seen in *Ubc9;Villin-Cre*^*ERT2*^*;Apc*^*f/+*^ animals, with medians of 2.5 and 6.5 polyps per mouse counted in *Ubc9*^*+/+*^ and *Ubc*^*+/−*^ mice, respectively. This is also in line with the improved overall survival of *Ubc9;Lgr5-IRES-EGFP-Cre*^*ERT2*^*;Apc*^*f/+*^ compared to *Ubc9;Villin-Cre*^*ERT2*^*;Apc*^*f/+*^ mice (compare Figs. 2b, 3a). Histological analysis again revealed no differences in morphology or general organization of lesions attributable to Ubc9 status (Fig. 3c). Altogether, these data show that loss of one *Ubc9* allele also enhances tumor formation in vivo in an *Apc*-*null* background limited to CBC cells.

### Transcriptomic analysis of Lgr5^+^ CBC cells reveals a cancer-related and pro-inflammatory basal state under Ubc9 haploinsufficiency

**Fig. 4:**
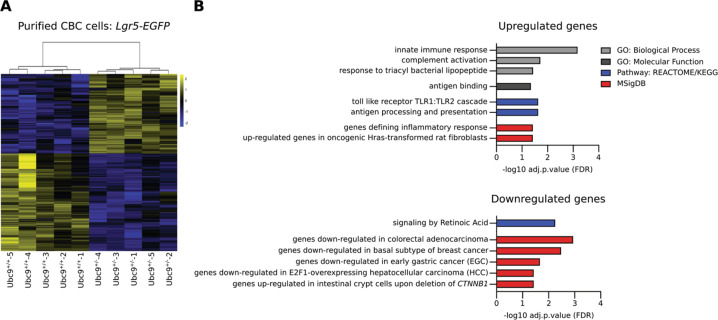
*Ubc9*^*+/−*^ CBC cells exhibit inflammatory and cancer-related transcriptomic signatures. **a** Heatmap of 205 differentially-expressed genes (|log2FC| > log2(1.5) and adj. *p* value (FDR) < 0.05) in Ubc9^*+/−*^ Lgr5-EGFP^+^ CBC cells from intestines of healthy *Lgr5-IRES-EGFP-Cre*^*ERT2*^ mice purified by FACS. *n* = 5 per group. **b** Gene Ontology (GO) and Pathway analysis of differentially-expressed genes upregulated (upper) and downregulated (lower) in Ubc9^*+/−*^ Lgr5-EGFP^+^ CBC cells with adjusted *p* value (FDR Benjamini-Hochberg) < 0.05. GO’s Biological Process, and pathways from KEGG and REACTOME and MSigDB databases were challenged.

Taking advantage of *Lgr5*-driven expression of EGFP in *Lgr5-IRES-EGFP-Cre*^*ERT2*^ mice, we obtained fluorescence-activated cell sorting (FACS)-purified Lgr5-EGFP^+^ cells derived from normal (*Apc*^*+/+*^) intestines from both *Ubc9*^*+/+*^ and *Ubc9*^*+/−*^ mice and performed a comparative analysis of their transcriptomic profiles (Fig. S4a). We found 205 DEGs (|log2FC| > log2(1.5) and adj. *p* value (FDR) < 0.05) in *Ubc9*^*+/−*^ Lgr5-EGFP^+^ cells, when compared to *Ubc9*^*+/+*^ Lgr5-EGFP^+^ cells, of which 92 and 113 were up- and downregulated, respectively (Fig. 4, Table S3).

Interrogation of curated gene expression databases, using as template up- and downregulated genes separately, now revealed a group of DEGs that have been associated with different types of cancers. Among the upregulated genes were genes reported to have increased expression in HRas-transformed rat fibroblasts (Fig. 4b, Tables S3, S4). Among downregulated DEGs in *Ubc9*^*+/−*^ Lgr5-EGFP^+^ cells, up to 17% were genes found to be repressed in early gastric cancer, colorectal adenocarcinoma, E2F1-overexpressing hepatocellular carcinoma and a subtype of basal breast cancer. Significantly, some downregulated genes (*Capn9, Pnliprp1, Pnliprp2, Cgref1, Cyp2u1, Aldh1a1*, and *Car4*) had also been found previously [29] to be upregulated in intestinal crypts upon deletion of β-catenin (*CTNNB1;* Fig. 4b, Tables S3, S4).

A further significant feature suggested by GO analysis of genes upregulated in *Ubc9*^*+/−*^ Lgr5-EGFP^+^ cells were top-hit signatures for the inflammatory response, innate immune response, genes involved in the response mediated by the membrane-bound toll-like receptors, complement activation and response to bacteria (Fig. 4b, Table S4). Genes from the above-mentioned signatures represent 25% of the upregulated DEGs found in *Ubc9*^*+/−*^ Lgr5-EGFP^+^ cells (Table S3), indicating that loss of one Ubc9 allele triggers a pro-inflammatory state in Lgr5^+^ CBC cells.

### *Ubc9*^*+/−*^ crypts harbor more Lgr5^+^ CBC cells

**Fig. 5:**
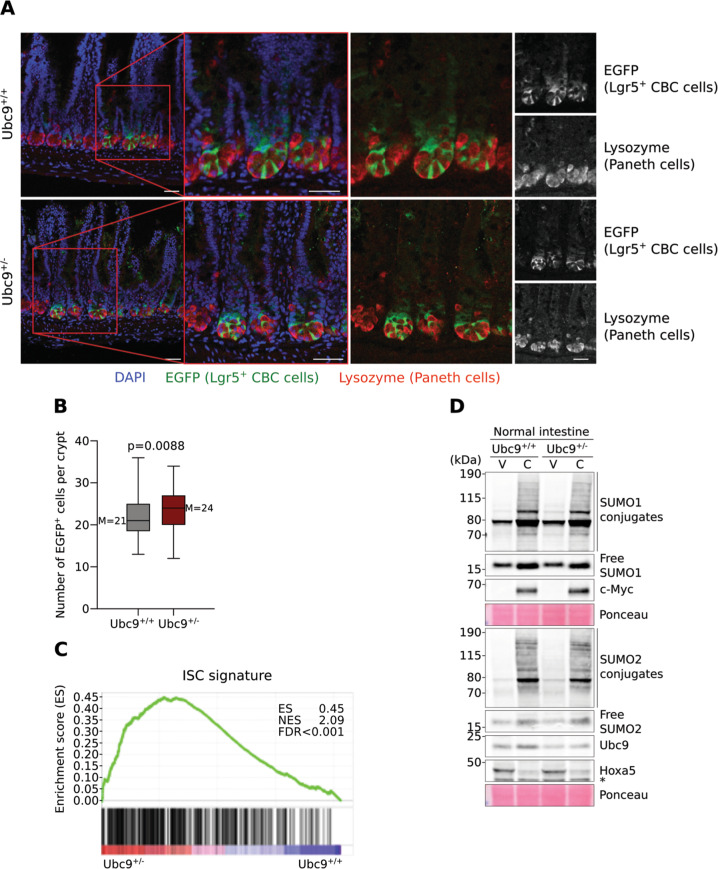
*Ubc9*^*+/−*^ crypts harbor more Lgr5^+^ CBC cells. **a** Confocal images of small intestinal crypts *Ubc9*^*+/+*^*;Lgr5-IRES-EGFP-Cre*^*ERT2*^ and *Ubc9*^*+/−*^*;Lgr5-IRES-EGFP-Cre*^*ERT2*^ mice in an *Apc*^*+/+*^ background. EGFP (Lgr5^+^ CBC cells, green), Lysozyme (Paneth Cells, red), and DAPI are shown. Scale bars, 50 μm. **b** Median (M, shown on graph) with min, 25 and 75 percentiles, and max values of Lgr5-EGFP^+^ CBC cells per crypt in small intestines as detected in (**a**). *n* = 137 crypts, 5 mice and 127 crypts, 5 mice for Ubc9^+/+^ and Ubc9^*+/−*^, respectively. Unpaired two-tailed t test was used. **c** Significant enrichment of ISC signature genes in *Ubc9*^*+/−*^ FACS-purified Lgr5^+^-GFP CBC cells analyzed by gene set enrichment analysis (GSEA). ES enrichment score, NES normalized enrichment score. **d** Western-blotting of SUMO1 and SUMO2 conjugates, Ubc9, c-Myc and Hoxa5 in intestinal villi (V)- and crypt (C)-enriched populations from healthy *Ubc9;Lgr5-IRES-EGFP-Cre*^*ERT2*^ mice. *Non-specific.

We next assessed whether Ubc9 haploinsufficiency could be associated with changes in intestinal crypts in *Lgr5-IRES-EGFP-Cre*^*ERT2*^ mice. In order to evaluate the localization of both Lgr5^+^ and Paneth cells, intestines from *Ubc9*^*+/+*^ and *Ubc9*^*+/−*^ mice were prepared for confocal microscopy and analyzed for EGFP and lysozyme, respectively. No differences were detected in the general architecture or localization of Lgr5-EGFP^+^ cells and Paneth cells within crypts from mice of both genotypes (Figs. 5a, S2a). Strikingly, blinded counting, however, revealed an elevated number of small intestinal Lgr5-EGFP^+^ CBC cells in *Ubc9*^*+/−*^ crypts (medians of 21 and 24 per crypt for *Ubc9*^*+/+*^ and *Ubc9*^*+/−*^, respectively; Fig. 5a, b). This trend was consistent all along the small intestine but was not seen in colonic crypts (Fig. S5a, b). Thus, Ubc9 haploinsufficiency leads to a modest (15%) but significant expansion of the cell compartment containing intestinal stem cells. In addition, comparison of our transcriptomic data with a previously identified Lgr5^+^ stem cell signature gene set [30] revealed a significant enrichment of the signature genes in the Ubc9^*+/−*^ CBC cells, suggesting a higher stemness state in mutant cells compared to the WT (Fig. 5c, Table S5).

### Enhanced sumoylation in intestinal crypts and polyps

Finally, we analyzed the sumoylation status in partially-purified villi and crypt-based cells from *Ubc9*^*+/+*^ and *Ubc9*^*+/−*^ animals. For this, washed, longitudinally-opened intestines were first scraped to release villi cells and then treated with EDTA to liberate crypt-based cells for analysis by western blotting. As expected, expression of markers for undifferentiated cells, c-Myc [31], and differentiated cells, Hoxa5 [32], was higher and lower, respectively, in crypt-enriched compared to villi-containing samples (Fig. 5d). In line with this observation, mRNA expression of β-catenin/Wnt pathway components known to be highly expressed in crypts, such as c-Myc, Smoc2 and Axin2 [30] was also found to be elevated (Fig. S5c, “C”), whereas expression of the differentiation marker Krt20 [33] was found to be higher in villi-enriched samples (Fig. S5c, “V”), thus also validating the proper separation of cell populations. Significantly, total free SUMO and SUMO conjugate levels were higher in crypt-enriched cell fraction (Fig. 5d, “C”), with, however, no clear differences between *Ubc9*^*+/+*^ and *Ubc9*^*+/−*^ samples, as also seen in whole intestine (Fig. 2a). Higher Ubc9 (see also Fig. 2e) and free SUMO levels were similarly observed in adenomatous polyps (P) when compared to normal tissue (N) in both *Ubc9*^*+/+*^ and *Ubc9*^*+/−*^ mice in an Apc-loss background (Fig. S5d). Remarkably, while this was accompanied by increased levels of SUMO conjugates in polyps from WT mice, we failed to detect such globally enhanced sumoylation in the tumors from *Ubc9*^*+/−*^ mice, indicating that, in the latter, Ubc9 levels appear limiting for efficient sumoylation. Together, these results point towards a greater global SUMO pathway activity in both the proliferative crypt compartment and in the dysplastic polyp tissue. Of note, this is consistent with findings in human colon adenocarcinomas that also exhibit elevated Ubc9 protein expression [34] and almost twofold increases in median transcript levels in tumors (*n* = 275) compared to non-tumor (*n* = 349) tissues (GEPIA database, http://gepia.cancer-pku.cn/index.html, queried for UBE2I (Ubc9)) [35].

## Discussion

Here we have shown that Ubc9 haploinsufficiency enhances tumorigenesis and lethality in Apc loss-driven mouse models of intestinal neoplasia. This surprising outcome is associated with a moderate but detectable (15%) increase in the number of Lgr5^+^ CBC cells, which, in addition, display a higher degree of stemness and cancer-, inflammation- and innate immunity-related gene expression signatures. We also found that Ubc9 haploinsufficiency enhances the proliferative capacities of transformed MEFs both in vitro and ex vivo. Together, these studies establish an as yet unappreciated tumor-suppressive activity for sumoylation.

A basic question emerging from these results is whether Ubc9 haploinsufficiency indeed translates into reduced cellular sumoylation, given that globally visible SUMO conjugate levels appear largely unaffected by loss of one *Ubc9* allele, notably in transformed MEFs and healthy intestine (Figs. 1b, 2a). It is difficult to imagine, however, that the observed phenotypes under haploinsufficiency result from compromised sumoylation-independent Ubc9 activity. Rather, we suggest the involvement of specific “under-modified” substrate conjugates, whose detection is masked here by other, abundantly-modified proteins in such global analysis. The identification of these specific substrates, although technically challenging, may thus shed important light on the pathways and molecular mechanisms involved. Moreover, while Ubc9 haploinsufficiency does not always reduce sumoylation visibly, it does reduce sumoylation capacity, as seen most clearly in polyps, a tumor state highly demanding in sumoylation (Fig. S5d). This indicates that different cell types make different demands on the SUMO system and, conversely, that they might therefore display different sensitivities to its reduction. The smaller polyp size under Ubc9 haploinsufficiency (Figs. S2e, S3b) indeed suggests that insufficient sumoylation capacity may be a handicap for tumor growth.

Why, in contrast, Ubc9 haploinsufficiency in transformed MEFs results in enhanced proliferation (Fig. 1) could be explained by both the reduction of an as yet uncharacterized growth-inhibiting function of sumoylation, that may also apply to Lgr5^+^ CBC cells (Fig. 5a, b), and to a reduced sensitivity to inhibition of these different cell types. In both cases our results show that reduced sumoylation capacity, when mild, is not necessarily associated with reduced proliferative capacity.

Our findings that *Ubc9*^*+/−*^ Lgr5^+^ CBC cells display a pro-inflammatory state under normal conditions (Fig. 4b and Tables S3, S4) are in line with sumoylation acting as a general repressor of the inflammatory response, as shown in myeloid cells [36]. Inhibition of Ubc9 has been shown to activate pro-inflammatory regulators, such as RelA, cFos, cJun, and IFN-γ in cultured HCT-8 epithelial cells, and to lead to decreased amounts of the anti-inflammatory IL-10 cytokine in primary intestinal epithelial cells [37]. Lower sumoylation rates, possibly due to enhanced SENP7 desumoylase activity, were also detected in patients with inflammatory bowel disease and were described as a prerequisite for the onset of inflammation in colons from mice with dextran sodium sulfate- (DSS-) induced colitis [37, 38]. Significantly, previous studies also indicate a link between increased CBC cell numbers, as seen here, and inflammation. For example, a small increase in the number of Lgr5^+^ CBC cells and a significant rise in BrdU^+^ proliferating cells per crypt in the large intestine has been described in mice administrated with the inflammatory agent DSS [39]. Similarly, intestinal organoids treated with low doses of pro-inflammatory IL-22 [40] or the chronic colitis-associated cytokine TNF-α [39], displayed a small but significant increase in the percentage of Lgr5^+^ CBC cells. These observations indicate that mild and non-pathogenic levels of inflammation may indeed promote CBC cell renewal, consistent with our results (Figs. 5a, b, S5a) and the known roles of inflammation in tumorigenesis and tissue regeneration [41, 42]. Conditions, such as Ubc9 haploinsufficiency that increases the number of stem cells together with promoting a pro-inflammatory state, would thereby also increase the number of cells with tumor-initiating potential, and hence tumorigenesis.

Recent findings showing that sumoylation represents a significant barrier to cell fate change [43, 44] may provide a further explanation for increased CBC cell number (Figs. 5a, b, S5a). While CBC cells dividing symmetrically at the crypt base represent the normal source of intestinal stem cells for tissue maintenance, mounting evidence indicates that during injury, lost stem cells in the crypt can be replenished from the “+4” “reserve” cell population [45], from committed secretory progenitor cells [46], from Paneth and enteroendocrine lineage precursors [47, 48] or from early enterocyte lineage (absorptive) cell precursors [49]. It would thus be interesting to determine if Ubc9 haploinsufficiency, like injury, increases such cellular plasticity leading to increased CBC cell number. Moreover, in this scenario, this may also contribute to a second function, enhanced inflammatory signaling, shown necessary for the conversion of such non-stem cells to cells with tumor-initiating potential [50].

Finally, our finding of a tumor-suppressive function of sumoylation appears at odds with previously established roles of sumoylation in cancer. Indeed, the pro-tumorigenic role of sumoylation is inferred from the enhanced levels of SUMO pathway enzymes, and hence, sumoylation dynamics, and from the accrued sensitivity to inhibition of global sumoylation seen in many cancer cells [reviewed in ref. 7]. It must be borne in mind, however, that, as shown here, Ubc9 haploinsufficiency increases the frequency of tumor initiation events, as seen by the increased number of polyps. Indeed, under normal (*Apc*^*+/+*^) conditions, reduced (i.e., haploinsufficient) Ubc9 activity appears sufficient to ensure cell functionality. However, when cells face stress, such as loss of Apc tumor suppressor activity, this may become a handicap for maintaining the controlled nonmalignant state, in line also with the observed cancer-related transcriptome signature.

Together, our findings support a view of sumoylation as a buffering system against stress. As such, this system must first be overcome (aided experimentally by Ubc9 haploinsufficiency) during the initiation phase of tumorigenesis, to be then rewired, or even enhanced, to serve in mechanisms that ultimately protect the cancerous state. Given the “druggability” of the SUMO pathway [16], deeper understanding of these dose- and cell type dependent, seemingly paradoxical consequences of targeting the SUMO system may have important therapeutic implications.

## Materials and methods

### Mice

*Ubc9*^*+/−*^ [10] and *Ubc9*^*f/−*^*;Rosa26Cre*^*ERT2*^ [23] mice have been described previously. *Ubc9*^*+/−*^*;Villin-Cre*^*ERT2*^*;Apc*^*f/+*^ mice were obtained by crossing *Ubc9*^*+/−*^ with *Villin-Cre*^*ERT2*^ mice [28] and then with *Apc*^f/+^ mice [27]. *Ubc9*^*+/−*^*;Lgr5-IRES-EGFP-Cre*^*ERT2*^ animals were obtained by crossing *Ubc9*^*+/−*^ with *Lgr5-IRES-EGFP-Cre*^*ERT2*^ mice [21]. *Ubc9*^*+/−*^*;Lgr5-IRES-EGFP-Cre*^*ERT2*^*;Apc*^*f/+*^ mice were created by crossing *Ubc9*^*+/−*^*;Lgr5-IRES-EGFP-Cre*^*ERT2*^ with *Apc*^*f/+*^ mice [27]. See Supplementary Information for details.

### Histology

Paraffin-embedded 4 µm sections were stained using H&E solution and blindly analyzed by a pathologist. See Supplementary Information for details.

### Cell culture and growth of transformed MEFs

MEFs were obtained from three *Ubc9*^*f/−*^*;Rosa26Cre*^*ERT2*^ or *Ubc9*^+/+^*;Rosa26Cre*^*ERT2*^ embryos at day E12.5 and pooled. Low-passage *Rosa26-Cre*^*ERT2*^ p53DN and HRAS^V12^-transformed MEFs were treated with 4-hydroxytamoxifen (4-OHT; Sigma-Aldrich) or ethanol (EtOH) as control for the indicated time for growth experiments and were also used for 2D plate colony formation (low density), foci formation (high density), and 3D soft-agar colony formation assays. See Supplementary Information for details.

### Homologous recombination reporter assay

HR was determined as described previously [51] using a DR-GFP reporter stably integrated into U2OS human osteosarcoma cells. See Supplementary Information for details.

### Western blotting

Protein extracts from cultured cells were obtained by lysing the cells directly in Laemmli buffer (Bio-Rad, Hercules CA, USA). Protein extracts from tissues were obtained by immersing the tissue in RIPA buffer in Lysing Matrix D-containing tubes (MP Biomedicals, Illkirch, France). See Supplementary Information for details.

### Flow cytometry and fluorescence-activated cell sorting (FACS)

Analysis of cell cycle and apoptosis in transformed MEFs was carried out using the Muse Cell Analyzer (Millipore, Hayward CA, USA) and the Muse Cell Cycle and Muse Annexin V and Dead Cell kits, respectively (Luminex, Austin TX, USA). Purification of *Lgr5-EGFP*^*+*^ CBC cells from normal non-treated *Ubc9;Lgr5-IRES-EGFP-Cre*^*ERT2*^ mice and preparation of villi- and crypt-enriched populations were performed adapting previously-described protocols [30, 52]. Single EGFP^high^, CD24^middle^, EpCam^positive^, and PI^negative^ CBC cells were sorted using MoFlo Astrios Cell Sorter (Beckman Coulter). See Supplementary Information for details.

### Quantitative PCR

RNA was purified using Trizol (Molecular Research Center, Cincinnati OH, USA) according to manufacturer’s recommendations. Quantitative real-time PCR was performed using the primer sets specified in Supplementary Table S6.

### Transcriptome profiling

RNA profiling was performed using GeneChip MoGene 2.0 ST Array (Applied Biosystems) and normalized and analyzed using open-source Bioconductor packages on R [53, 54]. Differentially-expressed genes (adj. *p* value (FDR) < 0.05 and |log2FC| > 1.5) were used to challenge curated databases [55–61]. For GSEA analysis, all differentially and non-differentially-expressed genes in FACS-purified EGFP^high^ CBC cells were queried against a previously published intestinal stem cell signature [30]. See Supplementary Information for details.

### Detection of Lgr5-EGFP^+^ CBC cells

Cryo-sections (50 μm thick) of intestines from *Ubc9;Lgr5-EGFP-IRES-Cre*^*ERT2*^ were analyzed for EGFP and Lysozyme expression by confocal microscopy and blindly analyzed using Fiji (ImageJ). See Supplementary Information for details.

### Statistics

Cell-based experiments were carried out with at least three biological replicates. Experiments involving mice were performed in at least six animals per group. Statistical analysis was performed with GraphPad Prism 8 software. See Supplementary Information for details.

## Supplementary information

Supplementary Methods and Legends

Supplementary Figure S1

Supplementary Figure S2

Supplementary Figure S3

Supplementary Figure S4

Supplementary Figure S5

Supplementary Table S1

Supplementary Table S2

Supplementary Table S3

Supplementary Table S4

Supplementary Table S5

Supplementary Table S6

## Data Availability

Transcriptomic data from this study were deposited in the GEO database (https://www.ncbi.nlm.nih.gov/geo/) under accession numbers GSE146106 (Fig. 4) and GSE146039 (Fig. S2).

## Code availability

Computer code can be accessed upon request.
