# Supplementary material for: An unanticipated tumor-suppressive role of the SUMO pathway in the intestine unveiled by Ubc9 haploinsufficiency
Source: Oncogene. 2020 Sep 18;39(43):6692–703. doi: 10.1038/s41388-020-01457-y (PMC7581512; doi:10.1038/s41388-020-01457-y)
Supplement: Supplementary file 1 — Supplementary Methods and Legends [file 41388_2020_1457_MOESM1_ESM.pdf]

Supplementary Materials and Methods and Figure Legends for:

An unanticipated tumor suppressive role of the SUMO pathway in the intestine unveiled by Ubc9 haploinsufficiency

Running title: Tumor-suppressive role of Ubc9

Ignacio López[1]*, Eleftheria Chalatsi[1, 2, 3]*, Saskia I. J. Ellenbroek[4], Alexandra Andrieux[1], Pierre-François Roux[1], Juan P. Cerapio[1, 5], Grégory Jouvion[6], Jacco van Rheenen[4], Jacob-S. Seeler[1]**, Anne Dejean[1]**.

[1] Nuclear Organization and Oncogenesis Unit, Equipe Labellisée Ligue Nationale Contre le Cancer, INSERM U993, Institut Pasteur, 75015 Paris, France.

[2] Sorbonne Université, Collège doctoral, 75005 Paris, France.

[3] Present address: Bio-Rad Laboratories France, Marnes-la-Coquette, France.

[4] Division of Molecular Pathology, Oncode Institute, The Netherlands Cancer Institute, Amsterdam, The Netherlands.

[5] Present address: Centre de Recherches en Cancérologie de Toulouse, Université de Toulouse, Toulouse, France.

[6] Experimental Neuropathology Unit, Institut Pasteur, 75015 Paris, France.

* These authors contributed equally to this work.

** Corresponding authors: Anne Dejean (anne.dejean@pasteur.fr)

Jacob-S. Seeler (jacob.seeler@pasteur.fr)

Supplementary Materials and Methods

Mice

Deletion of the floxed exon 14 of one *Apc* allele was induced by intraperitoneal injections of 4-OHT (Sigma-Aldrich) prepared at 10 mg/mL in corn oil with 4% ethanol (total of 0.1 mg per g of body weight) given three times at 12-hour intervals to 8-12 week-old male and female mice that were housed all together independently of their genotype. Polyp development was analyzed in blinded fashion 12 and 16 weeks after 4-OHT treatment for *Villin-* and *Lgr5-IRES-EGFP-Cre*^{ERT2} mice, respectively, by sampling the entire small intestine that was washed, longitudinally opened and scored and measured under the stereoscope. Normal tissue and polyp samples were excised and frozen in liquid nitrogen and kept at -80ºC until processing. For survival studies, mice were followed daily (in blinded fashion) and sacrificed when presenting disease symptoms or weight loss exceeded 20%.

Histology

Sampled intestines were prepared by the "Swiss Roll" technique (without longitudinal incision) and were incubated overnight in 10% formalin solution at pH 7 (VWR, Radnor PA, USA), then rinsed with water and stored in 70% ethanol (Sigma-Aldrich, St. Louis MO, USA) at 4°C until embedding in paraffin.

Cell culture

Primary and transformed MEFs (see below) were maintained in DMEM+GlutaMAX-I (Dulbecco's Modified Eagle Medium) medium (Gibco, ThermoFisher, Waltham MA, USA). Medium was supplemented with 100 Units/mL penicillin, 100 µg/mL streptomycin (Gibco) and 10% fetal calf serum (Gibco). Supplemented medium is hereafter referred to as DMEM. Cells, regularly tested for *Mycoplasma* contamination, were incubated in a humidified atmosphere at 37°C and 5% CO_2.

Viral production and infection of MEFs

4.5x10^5 Phoenix-Eco cells (HEK293T cells modified for the production of ecotropic retroviruses; ThermoFisher) were seeded in 10 cm dishes and incubated for 24 h. Medium was then changed for DMEM supplemented with 25 µM chloroquine (Sigma-Aldrich), and cells were transfected with calcium phosphate with 10 µg of retroviral plasmid construct DNA in 100 mM $CaCl_2$ in HBS (Hepes-buffered saline; Fluka) for 30 min at RT. Transfected cells were then incubated overnight at 37°C. The same day, 4.5x10^5 target cells to be infected were seeded in 10 cm dishes. The following two days, supernatant of Phoenix cells was harvested 4 times every 3 h, filtered through 0.45 µm, supplemented with Polybrene (8 µg/mL; Sigma-Aldrich) and applied to the target cells. Finally, puromycin (4 µg/mL; InvivoGen, San Diego, CA) or hygromycin (100 µg/mL; InvivoGen) were added for selection until complete mortality of non-infected control cells was observed. Retroviral constructs used for expression of dominant-negative p53 (*Trp53*, pBABE-hygro p53 DD; Addgene #9058), and active $HRas^{V12}$ (pBabe-puro Ras^{V12}; Addgene #1768), were a kind gift from Bob Weinberg.

Growth of transformed MEFs

MEFs were seeded at 10^5 cells per well into 6-well plates in 2 mL of DMEM, treated with 100 µg/mL of 4-hydroxytamoxifen (4-OHT; Sigma-Aldrich) prepared in 100% ethanol or ethanol (EtOH) as control, grown for the indicated time, fixed for 10 min in 10% formalin pH 7, washed and then stained with 0.01% crystal violet solution (Sigma-Aldrich) for 10 min at RT. After extensive washes, the crystal violet trapped in the cell membranes was solubilized with 1 mL of 10% acetic acid (Sigma-Aldrich) and its absorbance was measured at 590 nm to estimate the relative number of cells. Alternatively, MEFs were counted using Z1 Coulter Particle Counter (Beckman Coulter) according to manufacturer's instructions.

In 2D plate colony formation (low density) assays, 300 transformed MEFs were seeded in 10-cm dishes with DMEM that was changed every 3 days and were incubated for 14 days. MEFs were fixed with ice-cold methanol (Sigma-Aldrich) and then stained with 0.5% crystal violet. In foci formation (high density) assays, 10^6 transformed MEFs were seeded in 10-cm dishes with DMEM that was changed every 3 days and were incubated for 14 days.

MEFs were fixed with 4% PFA (Sigma-Aldrich) and stained with 0.5% crystal violet. In 3D soft-agar colony formation assays, $5x10^4$ transformed MEFs were seeded per well of a 6-well plate in 2 mL of 0.5% agar (Sigma-Aldrich) in DMEM medium on top of 2 mL layer of 1% agar in medium previously poured and solidified and were incubated for 4 weeks. 1 mL of medium was added on top and was changed every 3 days. Colonies were stained with 0.005% crystal violet.

Xenografts

10^6 low-passage *Rosa26-Cre*^{ERT2} p53 dominant-negative (DN) and HRas^{V12}-transformed Ubc9$^{+/+}$ and Ubc9$^{f/-}$ MEFs were resuspended in 200 µL of PBS and injected subcutaneously into the flanks of female athymic 7-8-week old nu/nu mice (Charles River Laboratories, Écully, France). Tumors were measured externally with a caliper every day and were allowed to grow until reaching the limit point set at 1000 mm^3. Tumor volume was determined using the equation $V = LxW^2x0.5$, where L is length and W is width.

Homologous recombination reporter assay

Homologous recombination was determined as described previously [1] using a DR-GFP reporter stably integrated into U2OS human osteosarcoma cells. DR-GFP reporter-expressing U2OS cells were a kind gift from Dr. Maria Jasin from the Memorial Sloan-Kettering, USA. pCBA-SceI expression plasmid (pCBA-SceI, Addgene #26477) was a kind gift from Sérgio F. de Almeida from Faculdade de Medicina da Universidade de Lisboa, Portugal. To asses HR efficiency, $1.5x10^5$ cells were seeded per well of a 6-well plate and reverse transfected with 2 µg of pCBA-SceI or empty vector (EV) using GeneJuice (EMD Millipore, Burlington MA, USA) according to manufacturer's instructions. After 24 h, 50 nM (total) siRNA targeting either firefly luciferase (GL2, 5'(CGUACGCGGAAUACUUCGA)TT3' and complementary oligo, Eurofins Genomics, Ebersberg, Germany), UBC9 (ON-TARGET plus Human UBE2I (7329) SMARTpool, Dharmacon, Lafayette CO, USA) or RAD51 (5888 GeneSolution, QIAGEN, Venlo, Netherlands) were transfected using HiPerFect transfection reagent (QIAGEN) following the manufacturer's recommendations. Forty-eight hours after siRNA transfection, cells were harvested for flow cytometry and RNA extraction followed by

RT and quantitative PCR.

Western-blotting

Laemmli buffer used to lyse cultured cells contains 2% SDS, 10% glycerol, 60 mM Tris-HCl pH 6.8 and 0.01% bromophenol blue. RIPA buffer [10 mM Tris-HCl pH 8, 1 mM EDTA, 0.5 mM EGTA, 1% Triton X-100, 0.1% SDS, 0.1% sodium deoxycholate, 140 mM NaCl] was supplemented with 10 mM N-EthylMaleimide and complete EDTA-free protease inhibitors (Roche, Basel, Switzerland) was used for tissue lysis in a FastPrep24 (MP Biomedicals) with 6 cycles of 30 seconds at maximum power, interspersed with 5 min incubation on ice. Extracts were later on sonicated and centrifuged at maximum speed for 5 min. Protein concentration in supernatants was measured using Pierce 660 nm protein assay supplemented with ionic detergent compatibility reagent (IDCR, ThermoFisher) following manufacturer's instructions. Equal amounts of proteins were loaded on 4-12% Bis-Tris Criterion (Bio-Rad) or NuPAGE 4-12% Bis-Tris (Invitrogen, Waltham, MA) gels for electrophoresis. Proteins were transferred to nitrocellulose membranes using the Trans-blot Turbo transfer pack and system (Bio-Rad). Equal loading of samples was assessed by Ponceau S staining (Sigma-Aldrich) after transfer or by staining with anti-β-actin antibody. When necessary to detect multiple protein species, identical sample amounts were re-deposited on the same or a separate gel, to be run, blotted and processed in parallel, each with loading controls (Ponceau S), as indicated. Membranes were blocked with either 5% low-fat milk or 3% BSA in PBS-Tween, incubated with primary antibodies overnight at 4ºC, followed by 1 h incubation with secondary antibodies coupled to DyLight-680 or -800 nm fluorophores (ThermoFisher) and scanned using the Odyssey infrared system (LI-COR, Lincoln NE, USA). Original TIFF files were transferred to ImageJ software and the entire image was inverted. Images were adjusted via Image>Adjust>Brightness/Contrast with no gamma adjustment to visualize differences and relevant parts were then transferred to Inkscape graphic program for figure assembly. At this stage, only proportional adjustments in image size were made and no other processing was performed.

Antibodies used were: primary rabbit monoclonal anti SUMO1 (1:750; Y299, Abcam, Cambridge, UK, ab32058), anti SUMO2/3 (1:500; 18H8, Cell Signaling Technology 4971,

Danvers MA, USA), anti c-Myc (1:1000; Y69, Abcam ab32072), mouse monoclonal anti SUMO2/3 (1:500; 8A2, Abcam ab81371), anti Ubc9 (1:500; BD Transduction Laboratories 610749, San Jose CA, USA), anti β−actin (1:5000; Sigma-Aldrich A1978), and rabbit polyclonal anti Hoxa5 (0.5 µg/mL; Abcam ab82645).

Flow cytometry and fluorescence-activated cell sorting (**FACS**)

For analysis of cell cycle and apoptosis in transformed MEFs, $2.5x10^4$ cells were seeded per well of a 6-well plate with 2 mL of DMEM and were incubated for 4 days. Cells were harvested, counted and stained using the Muse Cell Cycle and Muse Annexin V & Dead Cell kits, respectively (Luminex), according to manufacturer's instructions. 5000 events were considered. DR-GFP reporter-expressing U2OS cells were harvested and stained with 3 µM Propidium Iodide (PI, Invitrogen) on ice and GFP-expressing cells were counted using CytoFLEX Flow Cytometer (Beckman Coulter, Brea CA, USA) and analyzed with FlowJo (FloJo LLC, Ashland OR, USA). 25000 events were considered. For purification of *Lgr5-EGFP*$^+$ CBC cells from normal non-treated 8-12 week-old male and female *Ubc9;Lgr5-IRES-EGFP-Cre*^{ERT2} mice, intestines were washed with PBS and longitudinally opened on ice. Villi cells were gently scrapped-off using glass slides and crypt-containing tissues were incubated in 10 mM EDTA for 20 min on ice, followed by 2 washes with ice-cold PBS, a second incubation with 10 mM EDTA for 10 min and 2 more washes. Samples were vigorously agitated in every wash step. At this point, samples for villi- and crypt-enriched populations analysis were acquired. Crypt-enriched fractions were obtained by pooling together the last two wash fractions. For FACS, presence of crypts was checked under the microscope and crypt-containing fractions were pooled and filtered through a 70 µm nylon cell strainer (Corning, Corning NY, USA). Crypts were centrifuged and incubated with dissociation solution containing 0.3 U/mL dispase (Corning), 10 µM Y-27632 (Sigma-Aldrich) and 0.8 U/µL DNaseI (Sigma-Aldrich) at 37ºC for 30 min with pipetting cycles every 10 min. Single cells were then washed in 1% BSA in PBS, labeled with APC-coupled anti mouse CD24 (1:20, M1/69, BioLegend 101814, San Diego CA, USA), PE/Cy7-anti mouse EpCam (1:100, G8.8, BioLegend 118216) in 1% BSA in PBS for 20 min on ice. Then, cells were washed again in 1% BSA PBS, stained with 3 µM PI and filtered through a 40 µm nylon cell strainer (Corning)

prior to sorting into RNA later solution (Invitrogen) and kept at -80ºC until further processing.

Quantitative PCR

For expression analysis of markers in villi- and crypt-enriched and in FACS-purified cells, 2 µg and 50 ng of RNA was used, respectively. cDNA was generated using random hexamers and SuperScript IV reverse transcriptase (ThermoFisher) or iScript Reverse Transcription Supermix (Bio-Rad). Quantitative real-time PCR was performed with SYBR Green PCR master Mix (Applied Biosystems, Waltham MA, USA) or iTaq Universal SYBR Green Supermix (Bio-Rad) on a CFX96 PCR System (Bio-Rad).

Transcriptome profiling

Total RNA was purified from normal tissue and polyps from 4-OHT-treated *Ubc9;Villin-Cre^{ERT2};Apc$^{f/+}$* mice (3 mice of each genotype, 2 normal tissues and 2 polyps per mice) and from FACS-purified Lgr5-EGFP^{+} CBC cells from normal non-treated *Ubc9;Lgr5-IRES-EGFP-Cre^{ERT2}* mice (5 samples of each genotype) using TRIzol. RNA integrity was evaluated using 2100 Bioanalyzer and RNA 6000 Nano kit (Agilent, Santa Clara CA, USA) and RNA concentration was measured using Qubit RNA BR Assay (Invitrogen). Hybridization was performed on GeneChip MoGene 2.0 ST arrays and scanned on an Affymetrix GeneChip Scanner 3000 7G (Applied Biosystems). Data were normalized using the robust multi-array average (RMA) normalization approach implemented in the oligo package. Normalized datasets were collapsed using a Fred's Softwares microarray algorithm (collapse_genes-09); source code is available at: https://sites.google.com/site/fredsoftwares/products/collapse-microarray. In the case of Lgr5-EGFP^{+} CBC cells, data were finally corrected with the surrogate variable analysis (SVA) package [2] to remove sources of unwanted variation and consider batch effects. Principal component analysis and bi-clustering based on Pearson's correlation and Ward's aggregation criterion were used to check for consistency between biological replicates and experimental conditions at each step of the pre-processing. Normalized log-scaled data were further considered for differential analysis with limma [3]. Moderated F-statistics that combines the empirical Bayes moderated t-statistics for all contrasts into an overall test of significance for each probe were used to assess the

significance of the observed expression changes for each contrast. P-values were corrected for multiple testing using the FDR approach. Functional characterization was performed via Gene Ontology (GO) and gene set over-representation analysis using curated databases GO-Biological Process, GO-Cellular Component, GO-Molecular Function [4, 5], and pathways in REACTOME [6] and KEGG [7, 8], and MSigDB via the ToppGene Suite portal [9]. GSEA analysis was performed using the GSEA software from the Broad Institute (http://software.broadinstitute.org/gsea/index.jsp) [10], with 1000 gene_set permutations, No_Collapse of data, and the previously published intestinal stem cell signature [11].

Detection of Lgr5-EGFP^{+} CBC cells

Intestines of 8-12 week-old male and female *Ubc9;Lgr5-EGFP-IRES-Cre^{ERT2}* mice were flushed with PBS and fixed overnight at 4°C in PLP-buffer (containing PFA, $NaIO_4$, L-Lysine and P-buffer). After fixation, tissues were washed and placed in 30% sucrose overnight at 4°C. The following day the tissues were frozen using O.C.T. (Tissue-Tek, Sakura, Torrance CA, USA) and stored at -80°C. Cryo-slides were cut (50 µm thick), rehydrated for 10 min in 0.1 M Tris in PBS, permeabilized in 0.8% Triton X-100 in PBS for 10 min, stained with anti lysozyme antibody (1:2000; Dako A009902, Jena, Germany) and 0.5 µg/mL DAPI in PBS and mounted with Vectashield (Vector Laboratories, Burlingame CA, USA). Images were acquired on a Sp5 confocal microscope (Leica, Wetzlar, Germany) using a 25x water-immersion objective.

Statistics

The exact number of replicates are specified in the figure legends or figures. No statistical method was used to predetermine sample size, the experiments were not randomized and no data were excluded. The investigators were not blinded to allocation during experiments and outcome assessment in *in vitro* and xenografts experiments. Assessment of polyp occurrence, evaluation of limit points stablished for survival experiments, histopathological analysis and counting of EGFP^{+} cells in crypts were all carried out in blinded fashion. Data shown in column graphs represent the mean±SD, and data shown in box and whiskers graphs represent the median with Min, 25 and 75 percentiles, and Max values, as indicated in

the figure legends. When normality could be assumed, Student's *t*-tests was used to compare the difference between two groups, otherwise the Mann–Whitney test was chosen. p values are shown on figures when applicable. For western blotting, hematoxylin and eosin staining, immunofluorescence, gating protocol for FACS and cytometry, and stereoscopic images of polyps and tumors, representative images are shown. Each of these experiments was independently repeated at least three times.

Supplementary Figure Legends

Fig. S1. Characterization of MEFs obtained from *Ubc9*$^{f/-}$*;Rosa26Cre*^{ERT2} or *Ubc9*$^{+/+}$*;Rosa26Cre*^{ERT2} embryos and transformed by retroviral transduction of p53DN and HRAS^{V12}. **a** Growth at day 4 showing mean ± SD of n=4 biological replicates in technical triplicates. Unpaired two-tailed Mann-Whitney test was used. **b** Cell cycle distribution at day 4, as in a, of n=4 biological replicates in triplicates. **c** Apoptosis at day 4, as in b, of n=4 biological replicates in triplicates. **d** RT-qPCR for endogenous *p53* and *HRas*. Mean of n=2 biological replicates. **e** RT-qPCR for exogenous *p53DN* (CDS-del using primers flanking the deletion and plasmid/CDS using forward and reverse primers located on construct and CDS, respectively) and *HRas* (CDS using both primers on the CDS and plasmid/CDS using forward and reverse primers located on construct and CDS, respectively), as in d, with mean of n=2 biological replicates. **f** Western blotting of endogenous p53, endogenous and exogenous HRas, and Ubc9 expression in *Ubc9*$^{+/+}$ and *Ubc9*$^{f/-}$ *Rosa26-Cre*^{ERT2} MEFs at day 4.

Fig. S2. *Ubc9* haploinsufficiency favors polyp formation in *Villin-Cre*^{ERT2}*;Apc*$^{f/+}$ intestinal cancer mouse model. **a** Histological analysis of H&E-stained normal intestines from healthy *Ubc9*$^{+/+}$ and *Ubc9*$^{+/-}$ mice showing the general architecture of crypts. **b** Stereoscopic images of intestinal adenomas obtained in *Villin-Cre*^{ERT2}*;Apc*$^{f/+}$ mice 12 weeks after 4-OHT treatment. **c** Quantification of histological analysis shown in *Fig. 1d*. Intestinal dysplasia and tumors revealed by H&E staining of intestines from *Ubc9*$^{+/+}$ and *Ubc9*$^{+/-}$ *Villin-CreERT2;Apc*$^{f/+}$ as in **b**. Median with Min, 25 and 75 percentiles, and Max values, n=5 per group. **d** Same as *Fig. 1d* with higher magnification. **e** Polyp diameter (mm) 12 weeks after 4-OHT treatment measured through stereoscopic observation. Median with Min, 25 and 75 percentiles, and Max values. Unpaired two-tailed Mann-Whitney test was used, n=253 and n=608 for *Ubc9*$^{+/+}$ and *Ubc9*$^{+/-}$, respectively. **f** Heatmap showing 11 differentially expressed genes (|log2FC| > log2(1.5) and adj. pvalue (FDR) < 0.05) in polyps from *Ubc9*$^{+/-}$ compared to *Ubc9*$^{+/+}$ *Villin-CreERT2;Apc*$^{f/+}$ mice 12 weeks after treatment with 4-OHT. n=6, 2 different polyps per animal. **g** Same as in **f** for 31 differentially expressed genes comparing normal tissues. n=6, 2 different normal

tissues per animal. **h** Reduction of Ubc9 levels by siRNA does not lead to changes in homologous recombination (HR) in an artificial HR reporter system. Shown are percentages of GFP^+ U2OS cells stably expressing the DR-GFP HR reporter transfected with empty vector (EV) or SceI-coding plasmid along with indicated siRNAs. Mean ± SD, n=4 biological replicates in duplicates. **i** RT-qPCR for *UBC9* and *RAD51* in U2OS human cells stably expressing the DR-GFP HR reporter after transfection of SceI-coding plasmid along with siRNAs against firefly GL2, UBC9 or RAD51. Mean of n=2 biological replicates. **j** Gating strategy used in flow cytometry to quantify GFP^+ cells from **h**.

Fig. S3. Tumors and polyps formed in *Lgr5-IRES-EGFP-Cre*^{ERT2}*;Apc*$^{f/+}$ intestinal cancer mouse model. **a** Intestinal tumors at the time of sacrifice of 4-OHT-treated *Lgr5-Cre*^{ERT2}*;Apc*$^{f/+}$ mice. **b** Polyp diameter (mm) in *Lgr5-IRES-EGFP-Cre*^{ERT2}*;Apc*$^{f/+}$ mice 16 weeks after 4-OHT treatment measured through stereoscopic observation. Median with Min, 25 and 75 percentiles, and Max values. Unpaired two-tailed Mann-Whitney test was used, n=36 and n=64 for *Ubc9*$^{+/+}$ and *Ubc9*$^{+/-}$, respectively.

Fig. S4. *Ubc9*$^{+/-}$ CBC cells show enhanced inflammation state. **a** Gating strategy used to FACS purify single PI^-, $EpCam^+$, $CD24^{mid}$ and $EGFP^+$ mouse epithelial intestinal cells from healthy *Ubc9*$^{+/+}$ and *Ubc*$^{+/-}$ *Lgr5-IRES-EGFP-Cre*^{ERT2} *mice*. n=5 per group. **b** RT-qPCR for differentially expressed genes *Tlr1*, *App*, *Naip1* and *Pdk4* in cells purified as in **a**, using a different set of samples from the one used to obtain the transcriptome. Median with Min, 25 and 75 percentiles, and Max values, n=6 and n=7 for *Ubc9*$^{+/+}$ and *Ubc9*$^{+/-}$, respectively. Unpaired two-tailed Mann-Whitney test was used.

Fig. S5. *Ubc9*$^{+/-}$ crypts harbor more $Lgr5^+$ CBC cells. **a** Median with Min, 25 and 75 percentiles, and Max values of $Lgr5\text{-}EGFP^+$ CBC cells per crypt in different regions of the small intestine of healthy *Lgr5-IRES-EGFP-Cre*^{ERT2} mice. n=55, 35, 47 crypts in proximal, middle and distal regions, respectively, from n=3 *Ubc9*$^{+/+}$ mice. n=46, 26, 55 crypts in proximal, middle and distal regions, respectively, from n=4 *Ubc9*$^{+/-}$ mice. **b** As in **a** for colon crypts. n=45 and 54 crypts for *Ubc9*$^{+/+}$ (n=3 mice) and *Ubc9*$^{+/-}$ (n=4), respectively. Unpaired

two-tailed t test was used. **c** RT-qPCR for marker genes *c-Myc*, *Smoc2*, *Axin2*, *Krt20* in intestinal villi and crypt-enriched populations from healthy *Ubc9;Lgr5-IRES-EGFP-Cre*^{ERT2} mice. Mean ± SD of n=4 per group. **d** Western blotting with indicated antibodies of normal (N) and polyp (P) tissues from intestines of *Ubc9*$^{+/+}$ or *Ubc9*$^{+/-}$*-Villin-Cre*^{ErT2}*;Apc*$^{f/+}$ mice 12 weeks after 4-OHT treatment.

Supplementary References

1. Pierce AJ, Johnson RD, Thompson LH, Jasin M. XRCC3 promotes homology-directed repair of DNA damage in mammalian cells. Genes Dev. 1999;13:2633-2638.
2. Carvalho BS, Irizarry RA. A framework for oligonucleotide microarray preprocessing. Bioinformatics. 2010;26:2363-2367.
3. Ritchie ME, Phipson B, Wu D, Hu Y, Law CW, Shi W*, et al.* limma powers differential expression analyses for RNA-sequencing and microarray studies. Nucleic Acids Res. 2015;43:e47.
4. The Gene Ontology Consortium. The Gene Ontology Resource: 20 years and still GOing strong. Nucleic Acids Res. 2019;47:D330-D338.
5. Ashburner M, Ball CA, Blake JA, Botstein D, Butler H, Cherry JM*, et al.* Gene ontology: tool for the unification of biology. The Gene Ontology Consortium. Nat Genet. 2000;25:25-29.
6. Fabregat A, Jupe S, Matthews L, Sidiropoulos K, Gillespie M, Garapati P*, et al.* The Reactome Pathway Knowledgebase. Nucleic Acids Res. 2018;46:D649-D655.
7. Ogata H, Goto S, Sato K, Fujibuchi W, Bono HKanehisa M. KEGG: Kyoto Encyclopedia of Genes and Genomes. Nucleic Acids Res. 1999;27:29-34.
8. Kanehisa M, Furumichi M, Tanabe M, Sato Y, Morishima K. KEGG: new perspectives on genomes, pathways, diseases and drugs. Nucleic Acids Res. 2017;45:D353-D361.
9. Chen J, Bardes EE, Aronow BJ, Jegga AG. ToppGene Suite for gene list enrichment analysis and candidate gene prioritization. Nucleic Acids Res. 2009;37:W305-311.

10. Subramanian A, Tamayo P, Mootha VK, Mukherjee S, Ebert BL, Gillette MA*, et al.* Gene set enrichment analysis: a knowledge-based approach for interpreting genome-wide expression profiles. Proc Natl Acad Sci USA. 2005;102:15545-15550.
11. Muñoz J, Stange DE, Schepers AG, van de Wetering M, Koo BK, Itzkovitz S*, et al.* The Lgr5 intestinal stem cell signature: robust expression of proposed quiescent '+4' cell markers. EMBO J. 2012;31:3079-3091.
