# Supplementary figures and images for: An unanticipated tumor-suppressive role of the SUMO pathway in the intestine unveiled by Ubc9 haploinsufficiency
Source: Oncogene. 2020 Sep 18;39(43):6692–703. doi: 10.1038/s41388-020-01457-y (PMC7581512; doi:10.1038/s41388-020-01457-y)

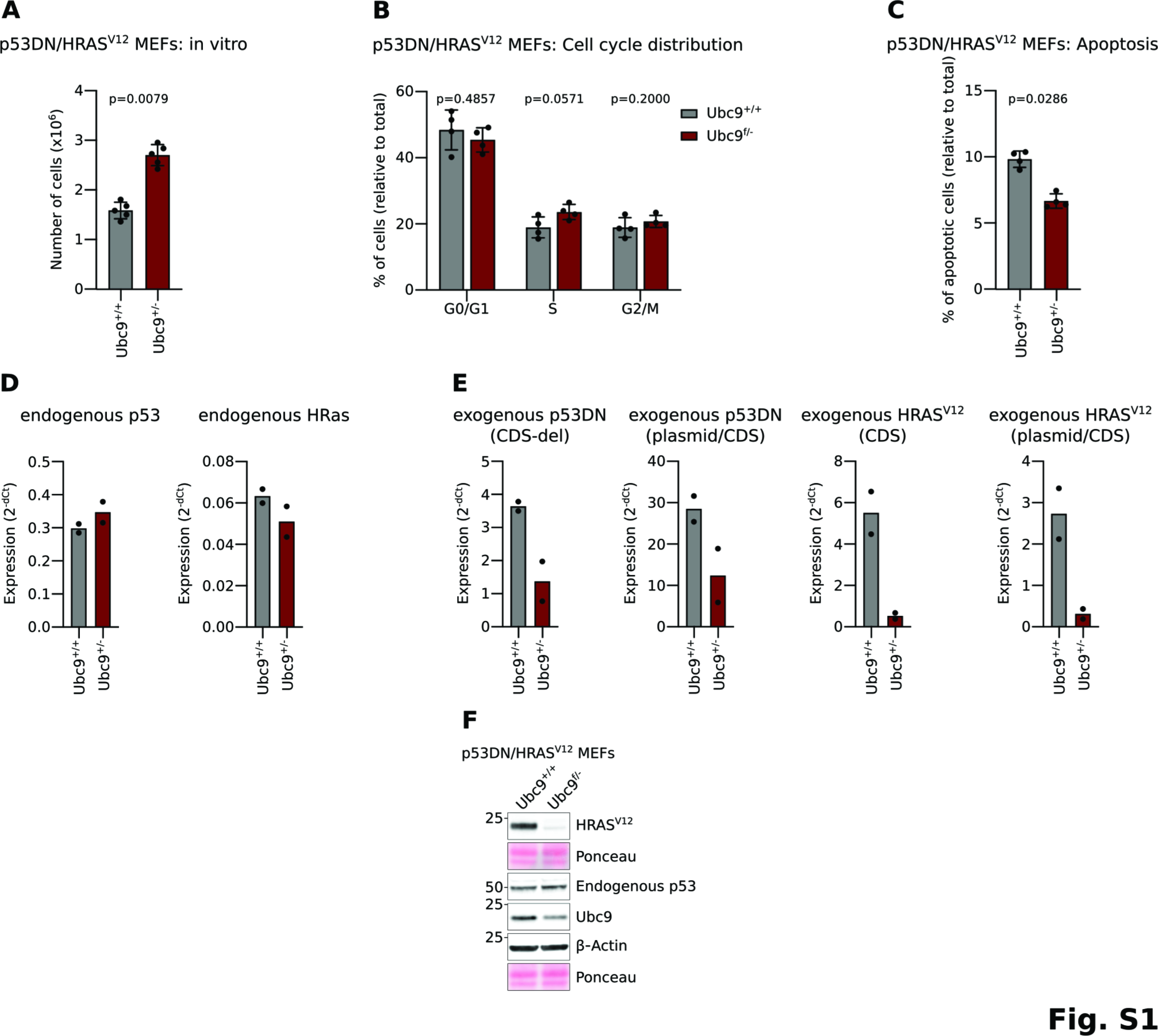

Supplement: Supplementary file 2 — Supplementary Figure S1 [file 41388_2020_1457_MOESM2_ESM.tif]

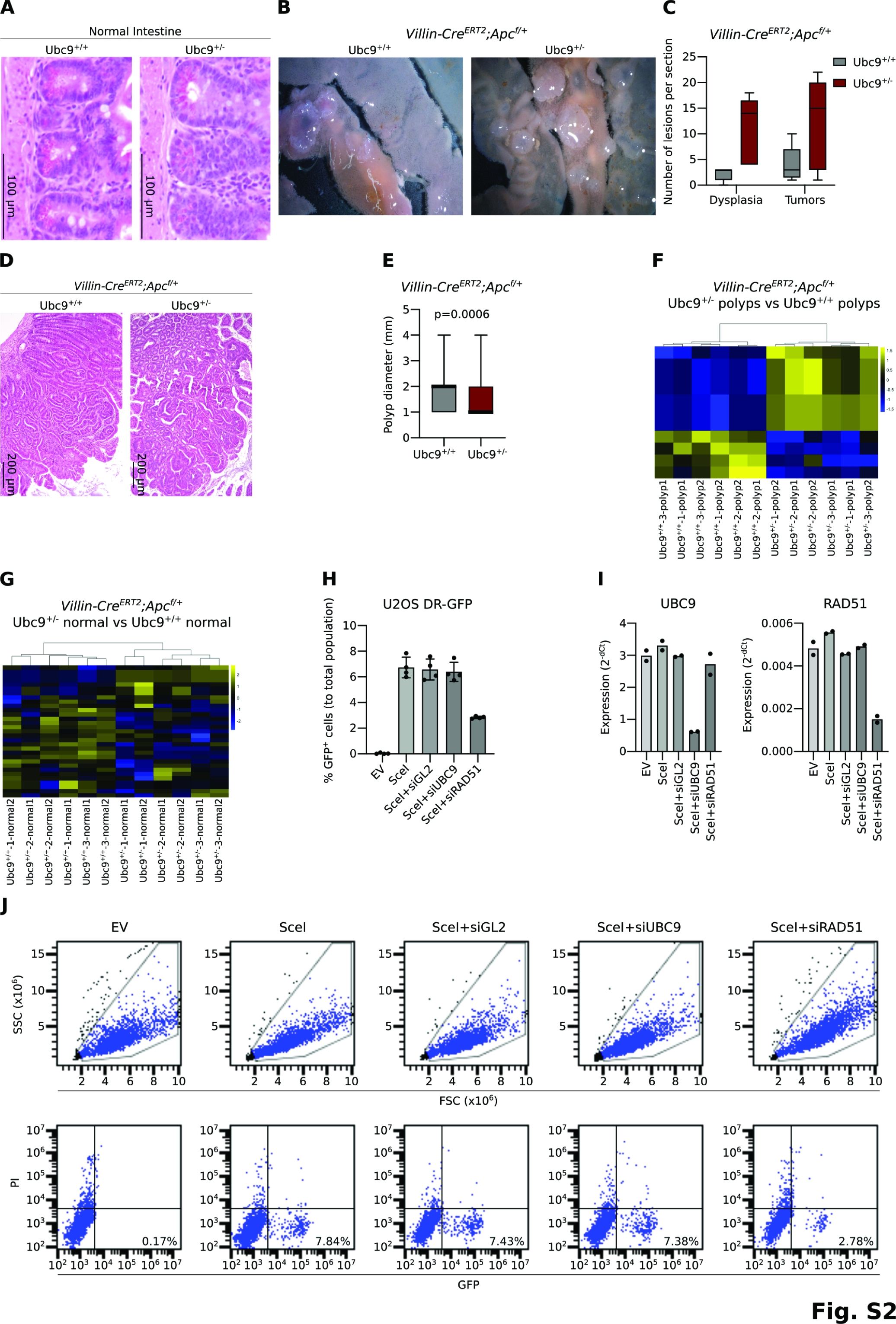

Supplement: Supplementary file 3 — Supplementary Figure S2 [file 41388_2020_1457_MOESM3_ESM.tif]

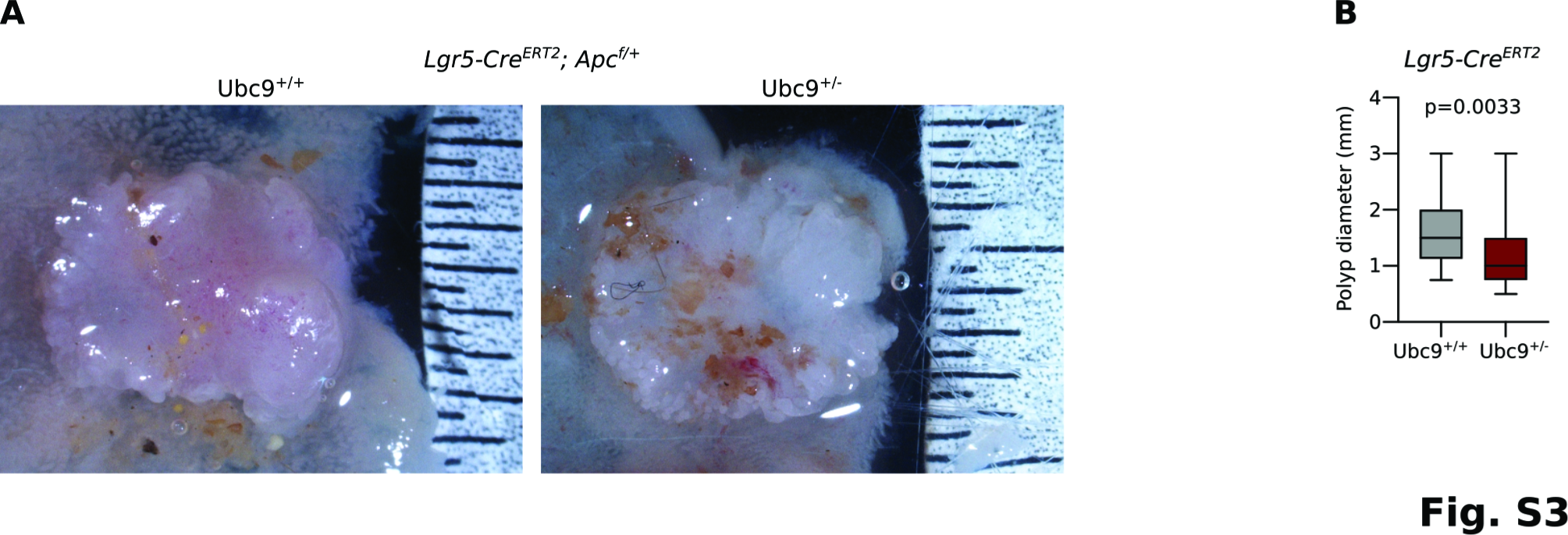

Supplement: Supplementary file 4 — Supplementary Figure S3 [file 41388_2020_1457_MOESM4_ESM.tif]

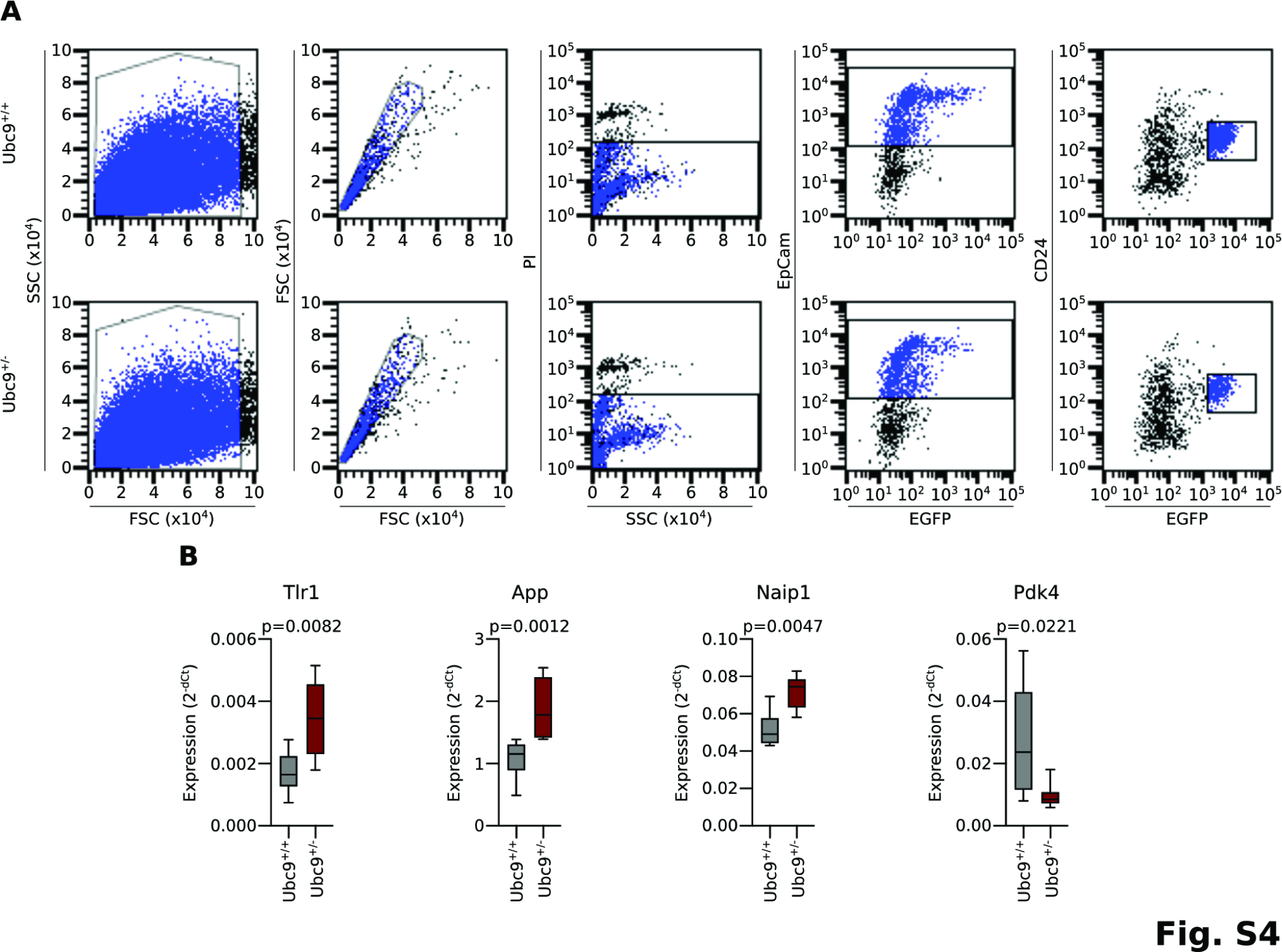

Supplement: Supplementary file 5 — Supplementary Figure S4 [file 41388_2020_1457_MOESM5_ESM.tif]

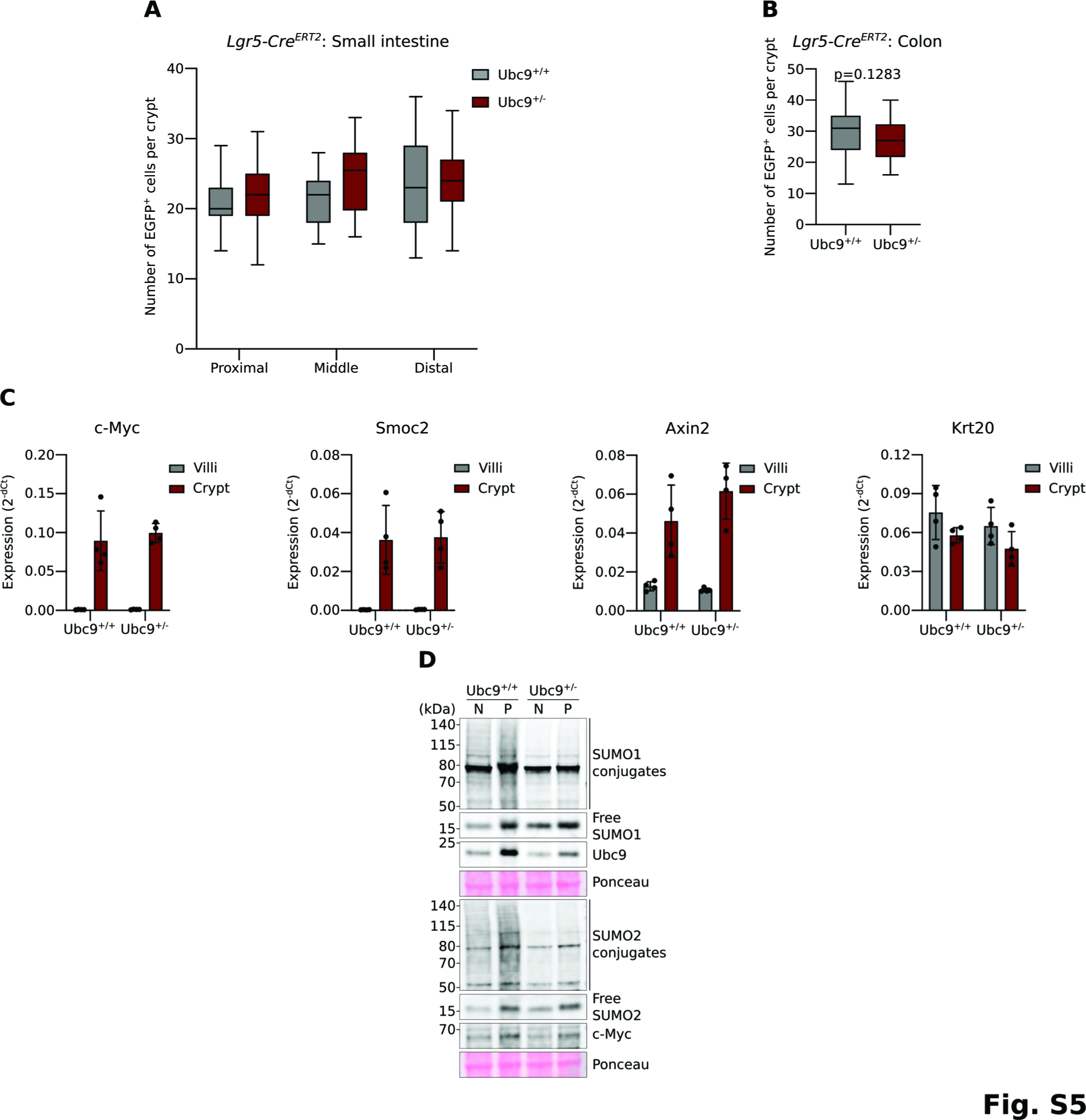

Supplement: Supplementary file 6 — Supplementary Figure S5 [file 41388_2020_1457_MOESM6_ESM.tif]
