# Supplementary material for: An unanticipated tumor-suppressive role of the SUMO pathway in the intestine unveiled by Ubc9 haploinsufficiency
Source: Oncogene. 2020 Sep 18;39(43):6692–703. doi: 10.1038/s41388-020-01457-y (PMC7581512; doi:10.1038/s41388-020-01457-y)
Supplement: Supplementary file 10 — Supplementary Table S4 [file 41388_2020_1457_MOESM10_ESM.pdf]

Supplementary table S4. Ontology terms obtained with DEGs found in FACS-purified *Lgr5*+ CBC cells from normal intestines of non-treated *Lgr5-IRES-EGFP-Cre*^{ERT2} mice.

Direction	Gene set	Database ID	Database	p-value	adj.p-val (FDR)	Hits in query list	Hits in Genome	Hit in query list
UP	innate immune response	GO:0045087	GO: Biological Process	8.52000E-08	6.73000E-04	13	745	Ighv1-18, App, Ighv1-63, Ighv1-34, Ighv5-12, Naip2, Cfi, Tmem173, Cd14, Tlr1, H2-Ab1, Csf1, Ighv1-4
UP	antigen binding	GO:0003823	GO: Molecular Function	4.10000E-07	1.89000E-03	8	248	Ighv1-18, Ighv1-63, Ighv1-34, Ighv5-12, Tap1, H2-Q6, H2-Ab1, Ighv1-4
UP	cell recognition	GO:0008037	GO: Biological Process	2.14000E-06	2.41000E-03	8	311	Nrp1, Ighv1-18, App, Ighv1-63, Ighv1-34, Ighv5-12, Hspa1b, Ighv1-4
UP	complement activation, classical pathway	GO:0006958	GO: Biological Process	2.82000E-05	1.94000E-02	6	215	Ighv1-18, Ighv1-63, Ighv1-34, Ighv5-12, Cfi, Ighv1-4
UP	Genes down-regulated in thymocytes: double negative versus CD4 [GeneID=920] single positive.	M6243	MSigDB C7: Immunologic Signatures (v6.0)	1.59800E-05	2.07400E-02	5	200	CSF1,TLR1,STING1,APP,DNAH8
UP	Genes most strongly down-regulated in kidney glomeruli isolated from TCF21 [GeneID=6943] knockout mice.	M6937	MSigDB C2: CGP Curated Gene Sets (v6.0)	1.64100E-05	2.07400E-02	3	31	ADM,HSPA1A,HSPA1B
UP	Mouse StemCell_vanderFlier09_134genes	19269367-TableS1	GeneSigDB	1.79500E-05	2.07400E-02	4	98	CLCA4,PLA2G5,APP,CFI
UP	Legionellosis	469200	Pathway: KEGG	2.74800E-04	2.34400E-02	3	55	CD14,HSPA1A,HSPA1B
UP	Antigen processing and presentation	83074	Pathway: KEGG	7.40300E-04	2.34400E-02	3	77	HSPA1A,HSPA1B,TAP1
UP	IRAK4 deficiency (TLR2/4)	1269158	Pathway: REACTOME	2.84400E-04	2.34400E-02	2	11	CD14,TLR1
UP	MyD88 deficiency (TLR2/4)	1269160	Pathway: REACTOME	2.84400E-04	2.34400E-02	2	11	CD14,TLR1
UP	Attenuation phase	1270424	Pathway: REACTOME	5.39900E-04	2.34400E-02	2	15	HSPA1A,HSPA1B
UP	Regulation of TLR by endogenous ligand	1427857	Pathway: REACTOME	6.16100E-04	2.34400E-02	2	16	CD14,TLR1
UP	ER-Phagosome pathway	1269197	Pathway: REACTOME	1.05600E-03	2.34400E-02	3	87	CD14,TLR1,TAP1
UP	Acyl chain remodelling of PS	1270065	Pathway: REACTOME	1.17600E-03	2.34400E-02	2	22	MBOAT1,PLA2G5
UP	HSF1-dependent transactivation	1270423	Pathway: REACTOME	1.28600E-03	2.34400E-02	2	23	HSPA1A,HSPA1B
UP	Toll Like Receptor TLR1:TLR2 Cascade	1269239	Pathway: REACTOME	1.36200E-03	2.34400E-02	3	95	CD14,TLR1,APP
UP	Toll Like Receptor 2 (TLR2) Cascade	1269238	Pathway: REACTOME	1.36200E-03	2.34400E-02	3	95	CD14,TLR1,APP
UP	MyD88:Mal cascade initiated on plasma membrane	1269237	Pathway: REACTOME	1.36200E-03	2.34400E-02	3	95	CD14,TLR1,APP
UP	Toll Like Receptor TLR6:TLR2 Cascade	1269240	Pathway: REACTOME	1.36200E-03	2.34400E-02	3	95	CD14,TLR1,APP
UP	Diseases associated with the TLR signaling cascade	1269157	Pathway: REACTOME	1.40100E-03	2.34400E-02	2	24	CD14,TLR1
UP	Diseases of Immune System	1269156	Pathway: REACTOME	1.40100E-03	2.34400E-02	2	24	CD14,TLR1
UP	defense response to bacterium	GO:0042742	GO: Biological Process	4.32000E-05	2.63000E-02	8	475	Ighv1-18, App, Ighv1-63, Ighv1-34, Ighv5-12, Naip2, Adm, Ighv1-4
UP	Antigen processing-Cross presentation	1269195	Pathway: REACTOME	1.71900E-03	2.69600E-02	3	103	CD14,TLR1,TAP1
UP	Acyl chain remodelling of PE	1270058	Pathway: REACTOME	2.04600E-03	3.02000E-02	2	29	MBOAT1,PLA2G5
UP	Viral RNP Complexes in the Host Cell Nucleus	1269122	Pathway: REACTOME	2.33500E-03	3.11100E-02	1	1	HSPA1A
UP	Activated TLR4 signalling	1269236	Pathway: REACTOME	2.35500E-03	3.11100E-02	3	115	CD14,TLR1,APP
UP	Apoptotic genes dependent on MAPK1 [GeneID=5594] and up-regulated in AML12 cells (hepatocytes) after stimulation with TGFB1 [GeneID=7040].	M1152	MSigDB C2: CGP Curated Gene Sets (v6.0)	3.71700E-05	3.22000E-02	2	6	TP53INP1,HSPA1B

UP	cellular response to triacyl bacterial lipopeptide	GO:0071727	GO: Biological Process	7.07000E-05	3.72000E-02	2	3	Cd14, Tlr1
UP	Selected genes up-regulated in Rat1Ras cells (fibroblasts) which were transformed by expression of an oncogenic activated form of HRAS [GeneID=3265] compared to the parental Rat1 cells.	M1265	MSigDB C2: CGP Curated Gene Sets (v6.0)	6.92500E-05	3.81400E-02	2	8	HSPA1A,HSPA1B
UP	Human Prostate_Nadiminty06_50genes_DownRegulated	16533764-Table2	GeneSigDB	7.00600E-05	3.81400E-02	3	50	CSF1,PXDN,TAP1
UP	Human Stomach_Nojima07_73genes	17297461-Table2	GeneSigDB	1.15100E-04	3.81400E-02	3	59	CSF1,HSPA1A,HSPA1B
UP	Mouse Lung_Rangasamy09_12genes	19286929-SuppTable2o	GeneSigDB	1.35600E-04	3.81400E-02	2	11	HSPA1A,HSPA1B
UP	Genes down-regulated in endothelial cells: IFNG [GeneID=3459] versus IFNG [GeneID=3459] and B. burgdoferi.	M6720	MSigDB C7: Immunologic Signatures (v6.0)	1.60800E-04	3.81400E-02	4	172	ADM,NOX1,CD14,TLR1
UP	Human Leukemia_Hamamura07_12genes	17895889-Table1a	GeneSigDB	1.62600E-04	3.81400E-02	2	12	HSPA1A,HSPA1B
UP	Genes up-regulated in apoptotic tissues (neuroepithelium) after MDM4 [GeneID=4194] knockout.	M5681	MSigDB C2: CGP Curated Gene Sets (v6.0)	1.75700E-04	3.81400E-02	4	176	ADM,TP53INP1,HSPA1B,TAP1
UP	Genes down-regulated in MEF cells (embryonic fibroblasts) after knockout of PML [GeneID=5371] and whose promoters were bound by MYC [GeneID=4609].	M1117	MSigDB C2: CGP Curated Gene Sets (v6.0)	2.23700E-04	3.81400E-02	2	14	ADM,HSPA1B
UP	Human Lung_Magda08_21genes	18593933-Table1a	GeneSigDB	2.23700E-04	3.81400E-02	2	14	HSPA1A,HSPA1B
UP	Down-regulated genes distinguishing between M1 (pro-inflammatory) and M2 (anti-inflammatory) macrophage subtypes.	M14515	MSigDB C2: CGP Curated Gene Sets (v6.0)	2.63700E-04	3.81400E-02	3	78	TLR1,HSPA1A,HSPA1B
UP	Genes up-regulated in comparison of untreated CD4 [GeneID=920] T cells at 0 h versus the cells treated with IL4 [GeneID=3565] and anti-IL12 at 6 h.	M4189	MSigDB C7: Immunologic Signatures (v6.0)	2.75500E-04	3.81400E-02	4	198	ADM,PXDN,TP53INP1,DENND5A
UP	Genes up-regulated in comparison of dendritic cells (DC) stimulated with LPS (TLR4 agonist) at 12 h versus DC cells stimulated with CpG DNA (TLR9 agonist) at 12 h.	M4005	MSigDB C7: Immunologic Signatures (v6.0)	2.80800E-04	3.81400E-02	4	199	CSF1,STING1,DENND5A,HSPA1B
UP	Genes down-regulated in Lung dendritic cell from Ad5 T424A hexon infection wildtype mice versus Lung dendritic cell from Ad5 T424A hexon inf IL-1R mice.	M9295	MSigDB C7: Immunologic Signatures (v6.0)	2.86200E-04	3.81400E-02	4	200	TP53INP1,TLR1,STING1,DNAH8
UP	Genes down-regulated in comparison of SP2 thymocytes versus SP4 thymocytes.	M5035	MSigDB C7: Immunologic Signatures (v6.0)	2.86200E-04	3.81400E-02	4	200	TLR1,APP,DNAH8,TAP1
UP	Genes up-regulated in comparison of dendritic cells (DC) stimulated with poly(I:C) (TLR3 agonist) at 0.5 h versus DC cells stimulated with CpG DNA (TLR9 agonist) at 0.5 h.	M3932	MSigDB C7: Immunologic Signatures (v6.0)	2.86200E-04	3.81400E-02	4	200	PLA2G5,DENND5A,FZD2,CD99L2
UP	Genes defining inflammatory response.	M5932	MSigDB H: Hallmark Gene Sets (v6.0)	2.86200E-04	3.81400E-02	4	200	ADM,CSF1,CD14,TLR1
UP	Genes down-regulated in dendritic cells: plasmacytoid versus cultured common progenitors treated by TGFB1 [GeneID=7040] for 4h.	M7833	MSigDB C7: Immunologic Signatures (v6.0)	2.86200E-04	3.81400E-02	4	200	TP53INP1,STING1,DNAH8,TAP1
UP	Genes down-regulated in comparison of SP1 thymocytes versus SP4 thymocytes.	M5030	MSigDB C7: Immunologic Signatures (v6.0)	2.86200E-04	3.81400E-02	4	200	TLR1,APP,DNAH8,TAP1
UP	Genes up-regulated in polymorphonuclear leukocytes 9h after infection by: S. aureus versus A. phagocytophilum.	M6207	MSigDB C7: Immunologic Signatures (v6.0)	2.86200E-04	3.81400E-02	4	200	ADM,TLR1,STING1,FZD2

UP	Genes down-regulated in comparison of untreated CD4 [GeneID=920] T cells versus those treated with IL1B [GeneID=3553] and IL6 [GeneID=3569].	M5600	MSigDB C7: Immunologic Signatures (v6.0)	2.86200E-04	3.81400E-02	4	200	MBOAT1,CYYR1,HSPA1A,HSPA1B
UP	Genes up-regulated in CD4 [GeneID=920] single positive cells: immature versus thymocytes.	M6248	MSigDB C7: Immunologic Signatures (v6.0)	2.86200E-04	3.81400E-02	4	200	TLR1,APP,HSPA1B,DNAH8
UP	Genes down-regulated in macrophages: untreated versus 24h after M. bovis BCG infection.	M7750	MSigDB C7: Immunologic Signatures (v6.0)	2.86200E-04	3.81400E-02	4	200	CABP2,CSF1,CD14,HSPA1A
UP	Toll Like Receptor 4 (TLR4) Cascade	1269234	Pathway: REACTOME	3.05200E-03	3.83000E-02	3	126	CD14,TLR1,APP
UP	Mouse Lung_Beisiegel09_262genes	19795415-Table1	GeneSigDB	3.26100E-04	4.13500E-02	4	207	CSF1,CD14,TLR1,TAP1
UP	Down-regulated genes in the canonical gene expression signature of the fibroblast core serum response (CSR) defined by the Stanford group.	M5793	MSigDB C2: CGP Curated Gene Sets (v6.0)	3.38300E-04	4.13500E-02	4	209	CSF1,TP53INP1,APP,SLC40A1
UP	Human Leukemia_Ge06_105genes	16249385-Table2	GeneSigDB	3.51600E-04	4.13500E-02	3	86	CD14,APP,HSPA1B
UP	Human Leukemia_Ruiz-Vela08_468genes	18032706-SuppTable3	GeneSigDB	3.58000E-04	4.13500E-02	5	387	NELL1,TLR1,FZD2,HSPA1A,CD99L2
UP	immune response-activating signal transduction	GO:0002757	GO: Biological Process	8.73000E-05	4.31000E-02	7	387	Ighv1-18, Ighv1-63, Ighv1-34, Ighv5-12, Cd14, Tlr1, Ighv1-4
UP	GPCR ligand binding	1269544	Pathway: REACTOME	3.66400E-03	4.37900E-02	5	455	ADM,NPSR1,APP,GRM4,FZD2
UP	endocytosis	GO:0006897	GO: Biological Process	9.30000E-05	4.46000E-02	8	531	Ighv1-18, App, Ighv1-63, Ighv1-34, Ighv5-12, Cd14, Adm, Ighv1-4
UP	signaling receptor binding	GO:0005102	GO: Molecular Function	1.94000E-05	4.48000E-02	16	1825	Ighv1-18, App, Pla2g5, Ighv1-63, Ighv1-34, Ighv5-12, Tap1, Retnlg, Eps8l1, Hspa1a, H2-Q6, Tlr1, Rarb, Csf1, Adm, Ighv1-4
DOWN	Human Breast_Muggerud06_271genes	16536878-SuppTable1	GeneSigDB	3.34900E-07	8.93800E-04	7	253	ACTG2,GSTM3,HOXB6,ALDH1A2,CNIH4,SCNN1A,SNCG
DOWN	Genes down-regulated in colorectal adenoma compared to normal mucosa samples.	M14791	MSigDB C2: CGP Curated Gene Sets (v6.0)	8.57300E-07	1.14400E-03	7	291	NAALADL1,PNLIPRP2,PDZK1,CHGA,GUCA2A,ZG16,CA4
DOWN	Genes down-regulated in basal subtype of breast cancer samles.	M4960	MSigDB C2: CGP Curated Gene Sets (v6.0)	3.72000E-06	3.31000E-03	9	701	CAPN9,PDZK1,GSTM3,HOXB6,AFP,KIAA1324,SCNN1A,ALDH1A1,SNCG
DOWN	Human Pancreas_Cavard09_22genes	19235837-Table3	GeneSigDB	7.23100E-06	4.82500E-03	3	21	PNLIPRP1,SYT3,CHGA
DOWN	Fat digestion and absorption	194385	Pathway: KEGG	9.17800E-05	5.61500E-03	3	41	PNLIPRP1,PNLIPRP2,FABP2
DOWN	Signaling by Retinoic Acid	1269630	Pathway: REACTOME	1.05900E-04	5.61500E-03	3	43	PDK4,ALDH1A2,ALDH1A1
DOWN	Human Breast_Muggerud06_232genes	16536878-SuppTable2	GeneSigDB	1.54200E-05	8.23200E-03	5	174	ACTG2,HOXB6,ALDH1A2,SCNN1A,SNCG
DOWN	Acylglycerol degradation	413388	Pathway: KEGG	2.46100E-04	8.69700E-03	2	11	PNLIPRP1,PNLIPRP2
DOWN	Digestion of dietary lipid	1270003	Pathway: REACTOME	6.03700E-04	1.60000E-02	2	17	PNLIPRP1,PNLIPRP2
DOWN	Down-regulated genes distinguishing between early gastric cancer (EGC) and normal tissue samples.	M15472	MSigDB C2: CGP Curated Gene Sets (v6.0)	4.87800E-05	2.17000E-02	6	366	PDK4,CHGA,PGC,ALDH1A2,KIAA1324,ALDH1A1
DOWN	RA biosynthesis pathway	1269631	Pathway: REACTOME	1.11400E-03	2.36200E-02	2	23	ALDH1A2,ALDH1A1
DOWN	Genes up-regulated in liver samples of liver-specific knockout of HNF4A [GeneID=3172].	M2193	MSigDB C2: CGP Curated Gene Sets (v6.0)	6.99400E-05	2.66700E-02	3	44	PDK4,GSTM3,KIAA1324
DOWN	Human Breast_Farmer05_269genes__basal_apocrine_luminal	15897907-SuppTable5	GeneSigDB	1.00700E-04	3.06600E-02	5	258	ACTG2,GSTM3,HOXB6,CNIH4,SCNN1A
DOWN	Genes up-regulated in liver tissue upon knockout of HNF1A [GeneID=6927].	M2397	MSigDB C2: CGP Curated Gene Sets (v6.0)	1.04500E-04	3.06600E-02	4	135	PDK4,FABP2,GSTM3,AFP
DOWN	Down-regulated genes from the set D (Fig. 5a): specific signature shared by cells expressing MLL-AF4 [GeneID=4297;4299] alone and those expressing both MLL-AF4 and AF4-MLL fusion proteins.	M527	MSigDB C2: CGP Curated Gene Sets (v6.0)	1.14900E-04	3.06600E-02	2	9	AFP,ALDH1A1

DOWN	Lipid digestion, mobilization, and transport	1270002	Pathway: REACTOME	1.91000E-03	3.37500E-02	3	115	PNLIPRP1,PNLIPRP2,FABP2
DOWN	Mouse EmbryonicStemCell_Abranches09_80genes	19621087-Table1b	GeneSigDB	1.60100E-04	3.75500E-02	3	58	PDZK1,GALR2,RASGRP2
DOWN	Genes up-regulated in intestinal crypt cells upon deletion of CTNNB1 [GeneID=1499].	M2342	MSigDB C2: CGP Curated Gene Sets (v6.0)	2.06000E-04	3.75500E-02	7	682	CAPN9,PNLIPRP1,PNLIPRP2,CGREF1,CYP2U1,ALDH1A1,CA4
DOWN	Genes down-regulated in hepatocellular carcinoma (HCC) induced by overexpression of E2F1 [GeneID=1869].	M15346	MSigDB C2: CGP Curated Gene Sets (v6.0)	2.14500E-04	3.75500E-02	3	64	PNLIPRP1,ALDH1A1,ZG16
DOWN	Human Breast_Turashvili07_201genes	17389037-Tab5	GeneSigDB	2.15700E-04	3.75500E-02	4	163	PDK4,PDZK1,ACTG2,FNDC1
DOWN	Human Liver_Capurso06_990genes	16728581-SuppTable1	GeneSigDB	2.34700E-04	3.75500E-02	8	932	PNLIPRP1,PNLIPRP2,ZNF521,ACTG2,FNDC1,CGREF1,SCNN1A,RASGRP2
DOWN	Genes up-regulated in MEF cells (embryonic fibroblast) with ELAVL1 [GeneID=1994] knocked out.	M2385	MSigDB C2: CGP Curated Gene Sets (v6.0)	2.47600E-04	3.75500E-02	4	169	C4orf3,HOXB6,ALDH1A1,SNCG
DOWN	Human Lung_Mikkonen10_416genes	20035825-TableS7a	GeneSigDB	2.47700E-04	3.75500E-02	5	313	PDK4,PDZK1,PGC,SCNN1A,RASGRP2
DOWN	Genes up-regulated during early stages of differentiation of embryoid bodies from V6.5 embryonic stem cells.	M2770	MSigDB C6: Oncogenic Signatures (v6.0)	2.53200E-04	3.75500E-02	4	170	SYT3,PDZK1,ZG16,CA4
DOWN	Genes which best discriminated between two groups of breast cancer according to the status of ESR1 and AR [GeneID=2099;367]: basal (ESR1- AR-) and luminal (ESR1+ AR+).	M5652	MSigDB C2: CGP Curated Gene Sets (v6.0)	3.15900E-04	4.43800E-02	5	330	CAPN9,PDZK1,GSTM3,KIAA1324,SCNN1A
DOWN	Human Breast_Schuetz06_101genes	16707453-SuppTable3	GeneSigDB	3.42900E-04	4.49600E-02	3	75	ACTG2,KIAA1324,SCNN1A
DOWN	Human Breast_Yau07_103genes	17850661-TableS4	GeneSigDB	3.70600E-04	4.49600E-02	3	77	ACTG2,GSTM3,ALDH1A1
DOWN	Human StemCell_Koide10_79genes	21152935-TableS2	GeneSigDB	3.70600E-04	4.49600E-02	3	77	GUCA2A,CGREF1,SNCG

Up- and downregulated DEGs in *Ubc9*$^{+/-}$ CBC cells (when compared to *Ubc9*$^{+/+}$) by applying |log2FC| > log2(1.5) and adj. p-value (FDR) < 0.05 as cutoff (from Supplementary table S3) were used separately to challenge the indicated databases.
